# Dual reinforcement of PEKK composites with hydroxyapatite and silicon nitride nanowires for enhanced mechanical, tribological, antibacterial, and osteogenic performance

**DOI:** 10.1186/s12951-026-04708-4

**Published:** 2026-06-26

**Authors:** Yuewen Xue, Lina Sun, Qinghua Tan, Tongtong Cui, Wanzhu Hao, Xia Luo, Ningyi Xian, Xijing He, Leilei Zhang, Ting Zhang

**Affiliations:** 1 Department of Orthopedics, The Second Affiliated Hospital of Xi’an Jiaotong University, Xi’an, 710000 P. R. China; 2 State Key Laboratory of Solidification Processing, Shaanxi Key Laboratory of Fiber Reinforced Light Composite Materials, Northwestern Polytechnical University, Xi’an, 710072 P. R. China; 3 Institute for Technical Chemistry and Polymer Chemistry (ITCP), Karlsruhe Institute of Technology (KIT), Engesserstraße 18, 76131 Karlsruhe, Germany; 4 Department of Rehabilitation, Beijing Rehabilitation Hospital of Capital Medical University, Beijing, 100043 P. R. China; 5 Department of Medical Imaging, The First Affiliated Hospital of Xi’an Jiaotong University, Xi’an, 710000 P. R. China; 6 Department of Dermatology, The First Affiliated Hospital of Xi’an Jiaotong University, Xi’an, 710000 P. R. China; 7 Department of Orthopedics, Xi’an International Medical Center Hospital, Xi’an, 710000 P. R. China

**Keywords:** PEKK composites, Hydroxyapatite, Silicon nitride nanowires, Tribological stability, Osseointegration

## Abstract

Polyetherketoneketone (PEKK) has attracted interest for load-bearing orthopedic implants due to its favorable elastic modulus and radiolucency; however, its biological inertness and weak interfacial bonding limit its clinical application. Here, we developed a dual-reinforced PEKK composite by incorporating hydroxyapatite (HA) particles and silicon nitride (SN) nanowires to improve its mechanical, tribological, antibacterial, and osteogenic properties. The ternary PEKK/HA/SN composite (PSH) exhibited tensile, compressive, and flexural strengths of 112.23, 241.36, and 201.48 MPa, increased from 23.04, 92.34, and 60.23 MPa for pure PEKK, respectively. Under dry sliding conditions, PSH showed a friction coefficient of 0.34 and a wear rate of 0.69 × 10^− 5^ mm^3^/N·m, corresponding to reductions of 30.6% and 24.2% compared to PEKK, respectively. In bovine serum, the friction coefficient and wear rate further decreased to 0.23 and 0.63 × 10^− 5^ mm^3^/N·m, with reductions of 45.2% and 25.9%, respectively. Furthermore, PSH exhibited antibacterial activity, with inhibition rates of 66.81% against *E. coli* and 75.25% against *S. aureus*, and reduced *S. aureus* biofilm biomass to 48.77 ± 6.51% of pristine PEKK. Biological assays showed improved osteoblast proliferation, osteogenic differentiation, and peri-implant bone formation in vivo. These findings suggest that the combined incorporation of HA and SN nanowires may provide a feasible strategy for improving the mechanical strength, tribological stability, antibacterial activity, and osteogenic performance of PEKK-based composites.

**Graphical Abstract:**

**Figure:**
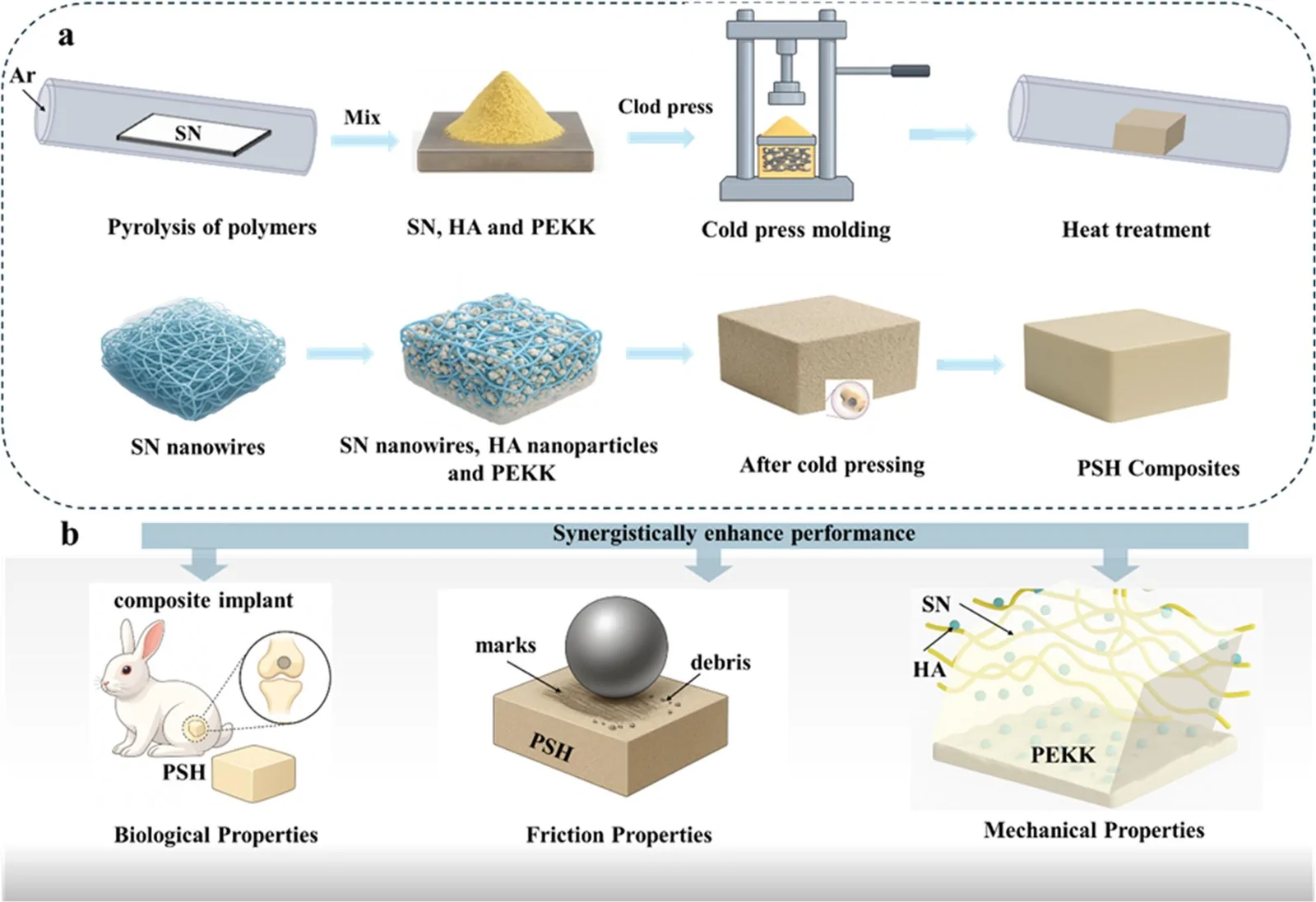

**Supplementary Information:**

The online version contains supplementary material available at https://doi.org/10.1186/s12951-026-04708-4.

## Introduction

Polyaryletherketone (PAEK) represents a class of high-performance semicrystalline thermoplastic polymers that have attracted increasing attention for orthopedic applications [1]. Compared with conventional titanium alloys, PAEK materials exhibit an elastic modulus closer to that of natural bone, which is favorable for alleviating stress shielding, while their inherent radiolucency enables artifact-free postoperative imaging [2, 3]. Quantitatively, titanium alloys typically exhibit an elastic modulus of approximately 110 GPa, whereas PAEK-based implants are commonly reported in the range of 3–4 GPa, which is closer to that of human cortical bone (approximately 7–30 GPa) [4–6]. Polyetheretherketone (PEEK) remains the most extensively investigated member of this polymer family. Since its approval by the U.S. Food and Drug Administration in 1998, PEEK has been widely used in spinal fusion, joint replacement, and maxillofacial reconstruction [7]. Despite this clinical success, its application in high-stress skeletal regions remains restricted by its limited load-bearing capacity [8]. While surface modifications and structural optimizations have been explored to address this limitation, their overall effectiveness remains constrained by the intrinsic mechanical characteristics of PEEK [8, 9]. Beyond inherent mechanical constraints, PAEK-based implants deployed in complex orthopedic conditions still face persistent challenges related to biological inertness, hydrophobicity, and insufficient multifunctional performance. Consequently, current material design strategies have evolved from single-function osteogenic enhancement to integrated approaches that simultaneously address bioactivity, antibacterial performance, and mechanical or tribological reliability [10, 11].

More recently, polyetherketoneketone (PEKK) has emerged as another important member of the PAEK family. Nevertheless, unmodified PEKK still faces several limitations in orthopedic applications. From a biological perspective, its bioinert surface leads to poor osseointegration with host bone, particularly under pathological conditions, thereby compromising long-term implant fixation [12, 13]. From a mechanical perspective, the long-term cyclic reliability of PEKK remains application-dependent, and its fatigue resistance can still be inferior to that of titanium in demanding fixation scenarios [14, 15]. In addition, neat PEKK faces tribological limitations under severe sliding conditions, where adhesive-fatigue wear and thermal-softening-related damage may occur, potentially leading to friction instability and debris generation [16]. Therefore, the development of PEKK-based implants for load-bearing bone repair requires a strategy that can simultaneously improve bioactivity, mechanical reliability, and tribological stability [17].

One widely investigated approach to address these limitations is the incorporation of functional fillers into the polymer matrix. Hydroxyapatite (HA), which closely resembles the main inorganic mineral phase of natural bone, is a representative bioactive filler. Previous studies have shown that HA incorporation can improve surface hydrophilicity and provide a supportive substrate for osteoblast adhesion, thereby supporting bone formation and osseointegration [4, 18, 19]. For example, Kang et al. reported that HA increased osteoblast proliferation and bone-implant bonding in PEEK-based composites [20]. However, the mechanical benefits of HA-filled polymer systems are often limited by particle agglomeration and weak interfacial bonding between HA and the polymer matrix, which may reduce reinforcement efficiency [21].

In contrast, silicon nitride (SN) nanowires provide advantages that are particularly relevant to mechanical reinforcement. SN is a high-performance ceramic with high fracture toughness, wear resistance, and load-bearing capability [22, 23]. When introduced as high-aspect-ratio nanowires, SN can form an interconnected reinforcing network within a polymer matrix [24]. Such a structure promotes stress transfer and increases energy dissipation through mechanisms including crack bridging, pull-out toughening, and crack deflection, thereby helping to alleviate the intrinsic brittleness of the host material [25–28]. In addition to these mechanical benefits, SN also exhibits biological advantages. In physiological environments, SN can gradually release soluble silicon species that participate in osteogenic regulation [29]. Previous studies have also shown that SN-containing surfaces support osteoblast differentiation and exhibit intrinsic antibacterial activity by suppressing bacterial adhesion and biofilm formation [29–33].

Despite these multifunctional characteristics, the osteogenic activity of SN remains lower than that of bone-mimicking HA [33–35]. Based on this consideration, we developed a dual-reinforcement strategy that combines HA nanoparticles and SN nanowires within a PEKK matrix. In this design, HA contributes bioactivity and a stiffness-modulating effect through its high modulus, whereas SN nanowires provide the dominant mechanical reinforcement together with antibacterial activity. Together, HA and SN form a dual-component reinforcement system that couples mechanical strengthening with biological function.

Herein, we report the fabrication and systematic evaluation of a ternary PEKK/HA/SN (PSH) composite. The material was characterized in terms of microstructure, mechanical properties, tribological performance, and surface characteristics. In addition, its biological performance was evaluated through protein adsorption, antibacterial assays, and in vitro osteoblast-related responses, followed by in vivo assessment in a rabbit femoral defect model. The results show that the combined incorporation of HA and SN improves the mechanical, tribological, antibacterial, and osteogenic performance of PEKK, indicating its potential relevance for load-bearing orthopedic implants.

## Materials and methods

**Fig. 1:**
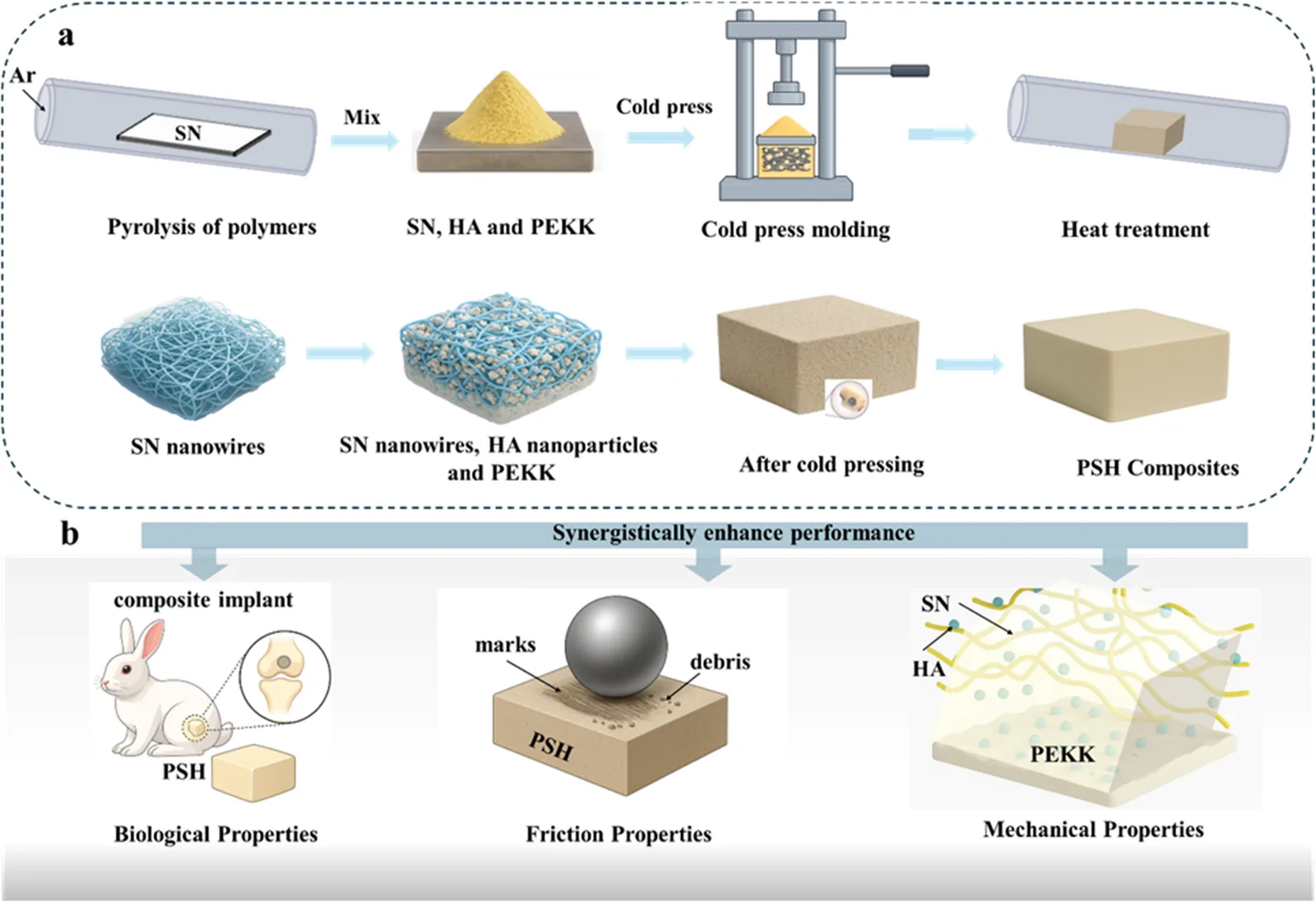
(**a**) Preparation process of PSH composites. (**b**) Combined improvement of mechanical, tribological, and biological properties

Figure 1a presents the preparation of PSH composites, while Fig. 1b illustrates the intended combined improvement in mechanical, tribological, and biological performance of the PSH composite.

### Preparation of SN nanowires

SN nanowires were synthesized by a polymer-derived ceramic route. The growth mechanism of SN is shown in Fig. S1. Polysilazane (Aladdin Reagent Co., Ltd., China) and ferrocene (Aladdin Reagent Co., Ltd., China) were mixed at a mass ratio of 9:1, while xylene (Aladdin Reagent Co., Ltd., China) and polysilazane were mixed at a volume ratio of 4:1. The mixture was ultrasonicated for 2 h until a homogeneous precursor solution was obtained. The precursor was transferred into an alumina crucible and placed in a horizontal tube furnace, followed by heat treatment at 1440 °C for 4 h under a flowing N₂ atmosphere. After furnace cooling to room temperature, SN nanowires were collected.

### Preparation of PEKK-based composites (PSN, PHA, PSH)

PEKK powder (Arkema Co., Ltd., China) was employed as the polymer matrix, while HA nanopowders (Aladdin Reagent Co., Ltd., China) and as-prepared SN nanowires were used as reinforcements. The PEKK/HA composite (PHA) was prepared by mixing PEKK and HA powders at a mass ratio of 2:1. The PEKK/SN composite (PSN) was prepared by mixing PEKK and SN nanowires at a mass ratio of 2:1. The PEKK/HA/SN composite (PSH) was prepared by mixing PEKK, HA, and SN nanowires at a mass ratio of 1:1:1. For each group, the mixed powders were loaded into a customized stainless-steel die (15 mm in diameter) and cold-pressed at 4 MPa to obtain green compacts. The green bodies were then subjected to a thermal consolidation step in an oxidation furnace at 350 °C for 20 min. This controlled consolidation process facilitated interparticle fusion of the PEKK phase, thereby improving compact integrity and reducing interfacial voids. Furthermore, this near-melting step promoted local densification and influenced the final crystallization behavior of the PEKK matrix during subsequent cooling [36–38]. Rather than serving as a conventional full-melt molding process, this step functioned as a controlled consolidation treatment for the cold-pressed green bodies [39, 40]. After naturally cooling to room temperature, the specimens were polished with 1500-grit abrasive paper, ultrasonically cleaned with ethanol and deionized water, and finally dried at 60 °C for 24 h. The resulting composites were designated as PHA, PSN, and PSH, respectively.

### Material characterization

The phase composition of the samples was identified by X-ray diffraction (XRD, D8 Advance, Bruker, Germany) using Cu Kα radiation (λ = 1.5406 Å), operated at 40 kV and 40 mA. The scanning range was set from 10° to 90° (2θ) with a step size of 0.02°. Fourier-transform infrared spectroscopy (FTIR, Nicolet iS50, Thermo Fisher Scientific, USA) was employed to investigate the chemical bonding and functional groups in the composites, with spectra recorded in the range of 4000 –400 cm^− 1^ at a resolution of 4 cm^− 1^. Raman spectroscopy (LabRAM HR Evolution, Horiba, France) was further conducted using a 532 nm excitation laser to probe the vibrational characteristics of the composite materials. The surface chemical states and elemental bonding configurations were examined using X-ray photoelectron spectroscopy (XPS, ESCALAB 250Xi, Thermo Fisher Scientific, USA) with Al Kα radiation as the excitation source. Survey spectra and high-resolution spectra were collected with pass energies of 160 eV and 40 eV, respectively, and all binding energies were calibrated to the C 1s peak at 284.6 eV. Surface topography and roughness parameters were analyzed by a three-dimensional (3D) optical profilometer (Contour GT-K, Bruker, USA), with measurements averaged from at least three different areas per specimen.

The microscopic morphology of the composites was characterized using a scanning electron microscope (SEM, JSM-6701 F, JEOL, Japan). Energy-dispersive X-ray spectroscopy (EDS), attached to the SEM system, was used to analyze the elemental distribution of the composites.

### Mechanical properties

Tensile tests were carried out on a universal testing machine (CMT54-30 kN, SANS, China) with samples of 10 × 15 × 5 mm (ISO 527) at a crosshead speed of 0.5 mm/min. Compression tests were performed on cylindrical samples (Φ 10 × 10 mm, ISO 604) at 0.5 mm/min, and three-point flexural tests were conducted on bars (30 × 10 × 3 mm, span = 24 mm, ISO 178) at 0.5 mm/min. All measurements were performed at room temperature (25 ± 2 °C) on 5 samples per group, and average values were reported. Representative photographs of the tensile, compressive, and flexural specimens are provided in Fig. S5.

### Nanoindentation measurements

Nanoindentation tests were carried out using a Nano Indenter G200 system (Agilent Technologies, USA) to determine the hardness and elastic modulus of the composites. Prior to testing, the specimen surfaces were polished to 1500-grit using SiC abrasive paper, ultrasonically cleaned, and dried, as described in Sect. "Preparation of PEKK-based composites (PSN, PHA, PSH)". A Berkovich diamond indenter tip was used under a maximum load of 100 mN. Continuous stiffness measurement (CSM) mode was applied to obtain load-depth curves. To avoid possible interference between adjacent plastic deformation zones, the spacing between neighboring indentations was maintained at not less than 100 μm. At least five valid indentations were performed at different positions on each sample, and the average values were reported.

### Biotribological performance

The friction and wear behavior of the composites was evaluated using a reciprocating friction and wear tester (HSR-2 M, Lanzhou Zhongke Kaihua, China). A stainless-steel ball (diameter: 4 mm, hardness > 60 HRC) was used as the counterbody, and the reciprocating stroke length was fixed at 5 mm. Each test was conducted for 30 min under a normal load of 380 N at room temperature. The load of 380 N was selected because it could generate stable and measurable wear within a practical testing window, while avoiding catastrophic failure of the polymer-based specimens. The test duration of 30 min was chosen to provide sufficient sliding cycles for the wear behavior to become representative and comparable among different material groups. A 25% fetal bovine serum (FBS) solution was used as the lubricant to simulate a protein-containing biological environment. The friction coefficient was continuously recorded during the test. The calculation formula for the wear rate is as follows:

$$\:{\upgamma\:}=\frac{\Delta\mathrm{V}}{\mathrm{F}\times\:\mathrm{L}}=\frac{\mathrm{A}}{2\mathrm{f}\times\:\mathrm{F}\times\:\mathrm{t}}$$

Where $\:{\upgamma\:}$ is the wear rate (mm^3^/N·m), $\:\Delta\mathrm{V}$ is the total wear volume (mm^3^), $\:\mathrm{L}$ is the total sliding distance (mm), $\:\mathrm{A}$ is the cross-sectional area of the wear mark (mm^2^), $\:\mathrm{f}$ is the frequency of friction test (Hz), $\:\mathrm{F}$ is the normal load (N), and $\:\mathrm{t}$ is the test time (s). Although the tests were performed at room temperature for comparative evaluation, the influence of physiological temperature and more complex in vivo loading conditions should be further investigated in future studies.

### Wettability and surface energy measurements

The surface wettability of the specimens was evaluated by static water contact angle measurement using a contact angle goniometer (JC2000DM, Shanghai Zhongchen Digital Technology Equipment Co., China). 5 µL of deionized water was gently placed onto the specimen surface at room temperature, and the contact angle was recorded after 5 s. For each sample, measurements were performed at 5 different positions, and the average value was reported. Surface free energy was calculated using the Owens-Wendt method based on the contact angles of deionized water and diiodomethane. Representative droplet images were collected simultaneously during the measurement process.

### Protein adsorption on the specimens

Protein adsorption on the specimen surfaces was evaluated using the Bicinchoninic Acid (BCA) Protein Assay Kit (New Cell & Molecular Biotech Co., Ltd, Suzhou, China) according to the manufacturer’s guidelines. Briefly, the samples were positioned in a 24-well plate, followed by the addition of 1 mL of 20 mg/mL bovine serum albumin (BSA, GlpBio Technology, USA) to each well, and incubation for 20 h at 37 ℃. Subsequently, the samples were gently rinsed twice with phosphate buffered saline (PBS) to eliminate any residual BSA. Adsorbed proteins were then eluted using 1 mL of 2% sodium dodecyl sulfate (SDS) with shaking for 3 h at 37 ℃. The collected eluent was analyzed for protein content via a BCA Protein Assay Kit, with absorbance readings taken at 562 nm.

### Antibacterial activity of specimens in vitro

Prior to the antibacterial assays, all specimens were immersed in 75 vol% ethanol overnight, air-dried under a laminar flow hood, and sterilized by autoclaving. Calibration curves of CFU/mL versus OD600 were established as follows. *Staphylococcus aureus* (*S. aureus*, ATCC 29213) and *Escherichia coli* (*E. coli*, ATCC 25922) were cultured overnight to the stationary phase, diluted 1:200 into 8 mL of fresh medium, and incubated at 37 °C with shaking. During incubation, 100 µL aliquots were sampled hourly into a 96-well plate (*n* = 3) for OD600 measurement, with parallel CFU/mL quantification by serial dilution plating.

For antibacterial evaluation, each specimen was incubated with 0.8 mL of the bacterial suspension (1 × 10^7^ CFU/mL) for 24 h at 37 °C. Post-incubation, the OD600 of the supernatant was measured in triplicate using a microplate reader (BioTek Synergy Neo2 Hybrid, Agilent, USA). To quantify adherent bacteria, specimens were aseptically transferred to tubes containing 5 mL of PBS and vortexed vigorously (HMS-350, HengAo Technology Co., China) to dislodge the cells. Finally, 50 µL of the serially diluted suspensions were plated on nutrient agar, incubated for 24 h at 37 °C, and evaluated for viable colony counts following the National Standard of China (GB/T 4789.2–2010), with pristine PEKK serving as the control.

Biofilm formation on the specimen surfaces was further evaluated by crystal violet staining against *S. aureus*. Sterilized specimens were placed in 24-well plates, each well containing 1 mL of *S. aureus* suspension (1 × 10^8^ CFU/mL) in brain heart infusion broth (BHI; Solarbio, China) supplemented with 1% (w/v) glucose. After static incubation at 37 °C for 48 h, the specimens were rinsed three times with sterile PBS to remove non-adherent bacteria. The adherent biofilms were fixed with methanol for 15 min, air-dried, and stained with 0.5% (w/v) crystal violet for 20 min at room temperature. Excess dye was removed by repeated rinsing with deionized water, and the stained biofilms were photographed. The bound dye was solubilized in 33% (v/v) acetic acid, and the absorbance of the eluent was measured at 570 nm using a microplate reader. Pristine PEKK served as the control for calculating relative biofilm biomass. Four independent replicates were performed for each group.

### Cell responses to specimens in vitro

MC3T3-E1 mouse preosteoblasts were obtained from Procell Life Science & Technology Co., Ltd. (China). Cells were routinely grown in α-Minimum Essential Medium (α-MEM, Gibco, USA) supplemented with 10% heat-inactivated fetal bovine serum (FBS, Cell-box, Changsha, China) and 1% penicillin-streptomycin solution (HyClone, USA), maintained at 37 °C in a humidified 5% CO₂ atmosphere (Thermo, USA). Prior to cell seeding, all specimens underwent sterilization via overnight immersion in absolute ethanol followed by autoclaving. The sterilized samples were then positioned in 24-well culture plates. For subsequent investigations into cellular adhesion, proliferative activity, and differentiation potential, MC3T3-E1 cells were plated directly onto the material surfaces at a density of 2 × 10^4^ cells per well. Throughout the culture period, half of the spent medium was replaced with fresh medium every 48 h.

#### Cell adhesion and morphology

To assess cellular adhesion, MC3T3-E1 cells were grown on the material surfaces for 24 h. Following this incubation period, the medium was aspirated and each sample was carefully washed twice with phosphate-buffered saline (PBS), then subjected to fixation with 4% paraformaldehyde solution for 12–16 h. The fixed specimens were subsequently stained in darkness with fluorescein isothiocyanate-phalloidin (FITC-phalloidin, Solarbio Science & Technology, Beijing, China) for 25 min and 2-(4-amidinophenyl)-6-indolecarbamidine dihydrochloride (DAPI, Solarbio Science & Technology, Beijing, China) for 5 min at ambient temperature. Cellular architecture was examined through a confocal laser-scanning microscope (A1R HD25, Nikon, Japan) to visualize nuclear and cytoskeletal organization. For ultrastructural analysis, the specimens underwent sequential dehydration in an ethanol gradient (30%, 50%, 75%, 90%, 95%, and 100% v/v), spending 10 min at each concentration, followed by air-drying at 25 °C before examination under a scanning electron microscope (SEM).

#### Cell proliferation and cell cycle

The cell proliferation assay was conducted using the high-sensitivity CCK-8 kit (KeyGEN BioTECH, China) following the manufacturer’s instructions after 1, 3, 5, and 7 days of culturing. Additionally, on Day 5, cells were harvested for flow cytometry to analyze the cell cycle. The cells were preserved in 70% cold ethanol at 4 °C overnight. Following a PBS wash, they were incubated with propidium iodide and RNaseA (Beyotime, Shanghai, China) for 30 min at room temperature. Flow cytometry was used to analyze the samples, and FlowJo software (FlowJo, LLC, Ashland, OR, USA) was employed to analyze the cell cycle distribution.

#### Quantitative real-time PCR analysis (qRT-PCR)

qRT-PCR analysis was performed at particular stages of proliferation and differentiation. Total RNA was isolated from MC3T3-E1 cells using RNAiso Plus (Takara, Osaka, Japan), and complementary DNA (cDNA) was synthesized using the PrimeScript™ RT reagent Kit with gDNA Eraser (Takara, Osaka, Japan). The Bio-Rad real-time PCR system (Bio-Rad, CA, USA) was used to conduct qRT-PCR analysis of genes related to the cell cycle and osteogenesis, with GAPDH serving as the reference gene for normalization. All experiments were performed in triplicate.

#### Western blotting

Cyclin protein levels were measured by extracting total cell protein on Day 5, while osteogenic protein levels were assessed by extracting total cell protein on Day 7. Equal amounts of protein samples were separated by 10% SDS-PAGE and then transferred to PVDF membranes. The membranes were treated with BSA, exposed to the primary antibody at 4 °C for 16 h, and then treated with the secondary antibody for 2 h at room temperature. The immunoblot bands were visualized with Ultra High Sensitivity ECL Kit (MedChemExpress, New Jersey, USA) on a BG-gdsAUTO 730 (Shanghai Baygene Biotechnologies Co., Ltd., Shanghai, China). Antibodies against Cyclin D1, Cyclin A2, Cyclin E1, PCNA, Bmp-2, OPN, OC, Col1a1, RUNX2 and β-actin were purchased from Abcam plc (Cambridge, UK). ImageJ (NIH, Bethesda, MD, USA) was used to quantify the intensity of the protein bands.

#### Cell differentiation

Following a 48-hour incubation period in 24-well plates to ensure cellular attachment and achieve approximately 70% confluence, the culture medium was replaced with osteogenic induction medium consisting of α-MEM supplemented with 10 nmol/L dexamethasone, 10 mmol/L β-glycerophosphate, and 50 mg/L ascorbic acid (Solarbio Science & Technology, Beijing, China). Alkaline phosphatase (ALP) activity was evaluated on days 7, 14, and 21 after induction using a commercial ALP detection kit (Beyotime, China). Concurrently, intracellular calcium deposition was quantified through a Calcium Colorimetric Assay Kit (abcam, UK).

For assessment of extracellular matrix mineralization, the cultured cells were fixed with 4% paraformaldehyde for 30 min at the termination of the culture period. After three PBS washing cycles, the fixed cells were treated with 1% Alizarin Red S solution (Solarbio Science & Technology, Beijing, China) for 30 min. Unincorporated dye was eliminated through multiple distilled water rinses prior to image acquisition. For quantitative analysis, the bound dye was solubilized with 500 µL of 1% cetylpyridinium chloride (CPC, MedChemExpress, New Jersey, USA) during 1 h of continuous agitation at 37 °C, and the absorbance of the resultant solution was recorded at 562 nm.

#### Immunofluorescence

On day 7 of osteogenic differentiation, the cells were gently rinsed with PBS for 3 min, followed by fixation with 4% paraformaldehyde for 20 min and permeabilization in 0.2% Triton X-100 for 30 min. Thereafter, the samples were washed and blocked with 5% normal goat serum in PBS for 30 min and incubated with primary antibodies against BMP-2 (Abcam, Cambridge, UK) at 4℃ overnight, followed by incubation with the secondary antibody CoraLite488-conjugated Goat Anti-Rabbit IgG (H + L) (Proteintech) for 1 h. The cells were then counterstained with 2-(4Amidinophenyl)-6-indolecarbamidine dihydrochloride (DAPI, Solarbio Science & Technology, Beijing, China) for 5 min, washed with PBS gently for 3 min, and observed using a confocal laser-scanning microscope (A1R HD25, Nikon, Japan).

### In vivo animal study

The Animal Research Committee of Xi’an Jiaotong University approved all animal experiments, which were conducted in accordance with the regulations for the Administration of Affairs Concerning Experimental Animals of China. Twenty-four male New Zealand rabbits, weighing 2–2.5 kg, provided by the Animal Experiment Center of Xi’an Jiaotong University, were included in the in vivo study. Bilateral femoral condyle defects were established in each animal, yielding a total of 48 femora. The rabbits were randomly and equally divided into three endpoint groups according to implantation duration (4, 8, and 12 weeks; 8 rabbits per time point). Within each time-point group, the 16 femora were randomly and evenly assigned to four material groups (PEKK, PHA, PSN, and PSH), with 4 femora in each group (*n* = 4). To reduce bias arising from individual variability, the same material was not implanted into both femora of the same rabbit.

#### Surgical procedure

The animals were fasted for 4 h preoperatively before surgery. Preoperative analgesia was provided by subcutaneous injection of vitacoxib (0.1 mg/kg, Orbiepharm, China). Anesthesia was induced by intravenous propofol (3–5 mg/kg, Petsun Therapeutics, China) and maintained with 1.5% isoflurane (BUCOLIC, China) via inhalation. Local infiltration of 0.5% lidocaine (BUCOLIC, China) was performed at the operative site prior to incision. Continuous respiratory support and vital sign monitoring were provided throughout the procedure using a dedicated animal ventilator system (Mindray, China).

After aseptic preparation, a 2.5 cm longitudinal incision was made along the medial aspect of the femoral condyle. Tissue layers were dissected to expose the underlying bone. A critical-sized (Φ 4 mm) defect was then created, into which the implants (sterilized as described in Sect. "Antibacterial activity of specimens in vitro") were placed. The surgical site was irrigated with sterile saline before layered closure of the wound and final antiseptic treatment.

Postoperative care included daily intramuscular injections of cefazolin sodium (10 mg/kg) for antibiotic prophylaxis and topical application of iodophor for wound disinfection, both maintained for 7 days. Following euthanasia at predetermined intervals (4, 8, and 12 weeks), femoral specimens were harvested and immediately preserved in 4% paraformaldehyde solution for subsequent histological and microstructural analyses.

#### Micro-CT analysis

Bone regeneration around the implants was evaluated by micro-computed tomography (micro-CT) imaging. The scanning procedure was conducted under the following operational parameters: 80 kV X-ray tube voltage, 70 µA beam current, and an isometric voxel size of 15 μm. A cylindrical volume of interest (VOI) was established surrounding each implant, extending from 1 mm beneath the growth plate to a distal boundary defined by 100 consecutive slices, while maintaining a radial offset of 100 μm from the implant interface. The acquired data sets were reconstructed and quantitatively analyzed using VGStudioMAX 3.0 software. The morphometric parameters included bone volume fraction (BV/TV), representing the proportion of mineralized tissue volume to total VOI volume; trabecular number (Tb. N); trabecular thickness (Tb. Th); and trabecular separation (Tb. Sp), indicating the average inter-trabecular distance.

#### Histological evaluation

Femoral condyle samples were embedded using methyl methacrylate (Sigma-Aldrich, St. Louis, MO, USA). The hard tissue sectioning system (Leica SP1600, Germany) was used to cut the samples into approximately 50 μm sections along the long axis of the implants. The tissue sections underwent histological staining with both Goldner’s trichrome (incorporating Weigert’s iron hematoxylin, Acid Ponceau, Orange G, and Light Green) and Methylene blue-acid fuchsin staining protocols before being subsequently examined under an optical microscope (OLYMPUS, Japan).

#### Bone formation fluorescence labeling

A sequential fluorescent labeling protocol was implemented to monitor osteogenic activity. Calcein green (30 mg/kg; Sigma-Aldrich, USA) and Alizarin complexone red (100 mg/kg; Sigma-Aldrich, USA) were administered by intramuscular injection at Week 4 and Week 8, respectively. Following histological sectioning, the spatiotemporal pattern of bone formation at the implant interface was visualized and assessed using laser confocal microscope (Leica TCS SP8, Germany).

### Statistical analysis

Quantitative data are presented as mean ± standard deviation (SD). Statistical analysis was performed using GraphPad Prism software (v9.0, GraphPad Software Inc., USA). For comparisons among four independent material groups at a single time point, ordinary one-way ANOVA followed by Tukey’s multiple-comparisons test was used. For datasets involving two factors, such as material group and culture time, two-way ANOVA followed by Tukey’s multiple-comparisons test was applied. When the assumption of homogeneity of variance was not met, Brown-Forsythe and Welch ANOVA followed by Dunnett’s T3 multiple-comparisons test was used. A value of *p* < 0.05 was considered statistically significant.

## Results and discussion

### Material characterization

#### Surface characterization

**Fig. 2:**
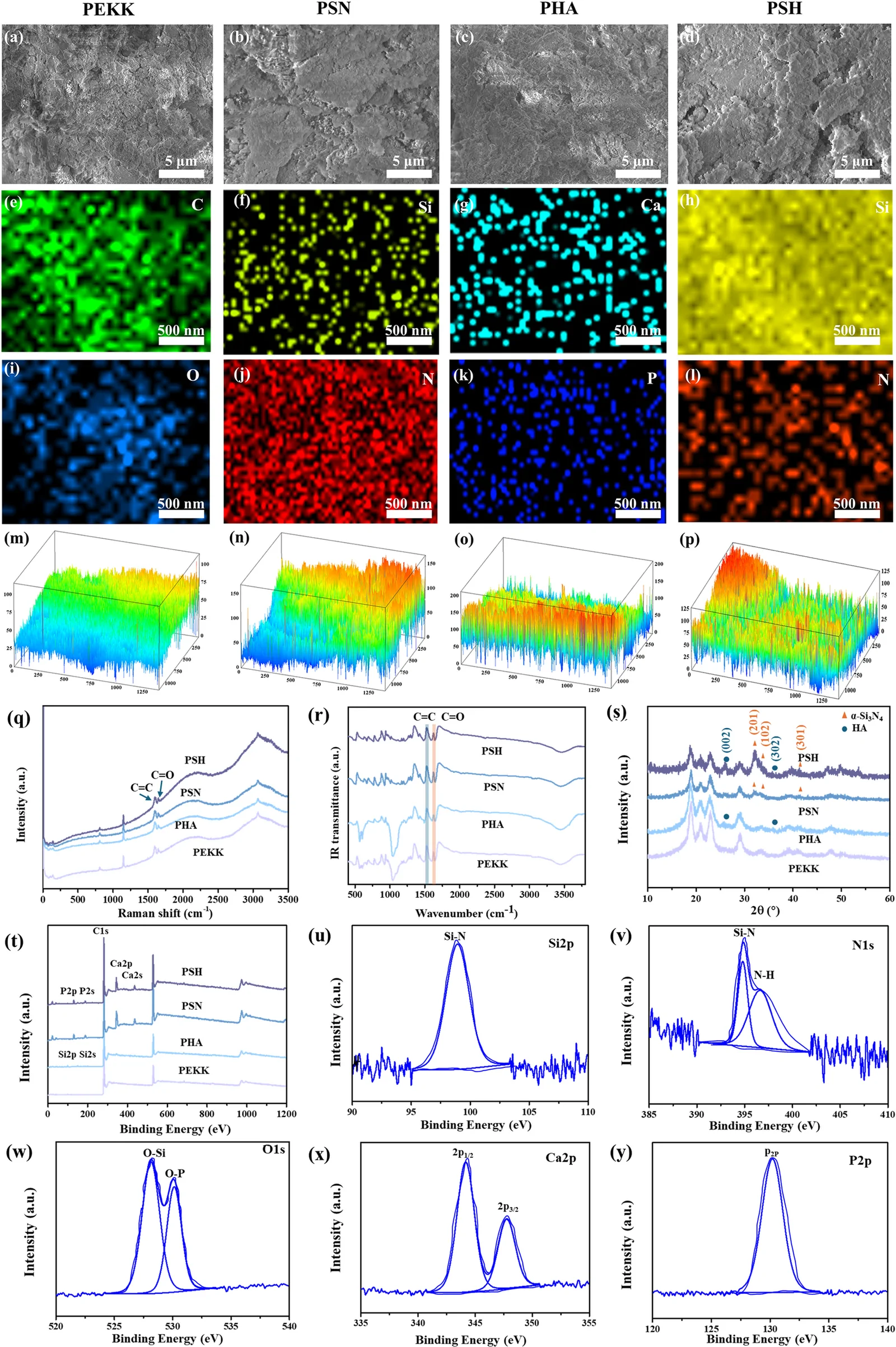
Characterization of PEKK-based composites: (**a-d**) SEM images of surface morphology for PEKK, PSN, PHA, and PSH; (**e-l**) EDS elemental mapping showing C, Ca, Si, O, P, and N distributions; (**m-p**) 3D surface profilometry of PEKK, PSN, PHA, and PSH; (**q**) Raman spectra; (**r**) FTIR spectra; (**s**) XRD patterns; (**t**) XPS spectra with peak fitting; (**u-y**) High resolution XPS spectra of PSH

Figure 2 shows the characterization of PEKK-based composites with different modifications. The SEM image indicates that pristine PEKK (Fig. 2a) exhibits a relatively smooth and uniform surface. After the incorporation of HA (Fig. 2c), the surface becomes rougher with more irregular features. Subsequently, SN was introduced into the PEKK matrix. The SN nanowires exhibited a 3D interconnected network with smooth surfaces; the corresponding TEM, XRD, and EDS characterization (Fig. S2 and Fig. S3) was consistent with pure α-Si_3_N_4_ and uniform elemental distribution. PSN (Fig. 2b) and PSH (Fig. 2d) exhibit rough surfaces due to the incorporation of HA and SN networks into PEKK, which increases the surface irregularities. This roughened surface morphology may provide additional anchoring sites for cell attachment and facilitate subsequent cell spreading and osteogenic differentiation. The EDS elemental mappings (Fig. 2e-l) showed uniform distribution of Ca, P, and O in PHA and uniform distribution of Si and N in PSN and PSH, consistent with the presence of HA and SN within the corresponding composites. The 3D surface profilometry **(**Fig. 2m-p, and Fig. S6) reveals that pristine PEKK has a relatively smooth and flat surface, whereas the modified composites exhibit increased surface roughness and height variations, with the PSH sample showing more evident topographical variations. Figure 2q-y illustrates the structural and chemical state analysis of PEKK-based composites. All samples exhibit characteristic Raman bands of the aromatic PEKK backbone at ~ 1590 cm^− 1^ (C = C stretching) and ~ 1640 cm^− 1^ (C = O stretching), indicating that the molecular structure of PEKK is retained after modification (Fig. 2q). The Raman spectra of PHA, PSN, and PSH are similar to those of PEKK, suggesting no detectable chemical decomposition or structural disruption occurred during the modification process. However, the gradual increase in background intensity and the broadening of Raman bands in PSH may reflect changes in the local interfacial microenvironment and possible inorganic-organic coupling within the composite, although direct interfacial bonding requires further characterization. In the FTIR spectra (Fig. 2r), all samples exhibit the characteristic absorption bands of the PEKK matrix, including the aromatic C = C stretching at ~ 1590 cm^− 1^ and the carbonyl (C = O) stretching at ~ 1640 cm^− 1^, confirming that the polymer backbone is well preserved. For the PHA composite, an additional broad absorption around 560 cm^− 1^ is attributed to the P–O bending vibration of HA, indicating the presence of HA in the composite. In PSH, a Si–N stretching band near 850 cm^− 1^ is detected, supporting the incorporation of SN. The XRD patterns (Fig. 2s) show characteristic diffraction peaks of PEKK at 18.3° and 22.4° for PEKK, while distinct reflections appear in PHA at 25.9° and 35.4°, which correspond to the (002) and (302) crystal planes of HA. In PSN, distinct reflections at 31.0°, 34.6°, and 42.3° correspond to the (201), (102), and (301) crystal planes of α-Si_3_N_4_. The PSH composite exhibits both HA and SN diffraction peaks, indicating the coexistence of these crystalline phases. The XPS survey spectrum of PSH reveals characteristic peaks of C, N, O, Ca, and P (Fig. 2t), with the high-resolution spectrum displaying Ca 2p and Ca 2s peaks located at 346.4 eV and 438.6 eV, respectively. Meanwhile, the P 2p and P 2s peaks at 133.3 eV and 189.9 eV are attributed to the phosphate species in the HA phase. The high-resolution Si 2p spectrum of the PSH composite (Fig. 2u) shows a dominant peak centered at 98.2 eV, which corresponds to the Si–N bonding characteristic of SN, indicating that the SN phase was retained within the PSH composite. The N 1s spectrum of PSH (Fig. 2v) shows two peaks at 395.6 eV and ~ 398.1 eV, assigned to Si–N and N–H bonds, respectively, confirming the presence of SN in the PSH matrix. The O 1s spectrum of PSH (Fig. 2w) can be deconvoluted into two peaks located at ~ 527.8 eV and ~ 531.5 eV, corresponding to O–Si and O–P bonds, respectively, which indicates the presence of HA in the PSH composites. For the PSH composite, the Ca 2p spectrum exhibits two distinct peaks at ~ 344.1 eV and ~ 349.4 eV, corresponding to the Ca 2p_1/2_ and Ca 2p_2/3_ states of HA (Fig. 2x). In addition, the P 2p spectrum shows a characteristic peak at ~ 130.2 eV, which is attributed to the PO_4_^3−^ group (Fig. 2y). These findings indicate the presence and chemical stability of HA and SN within the PSH matrix.

**Fig. 3:**
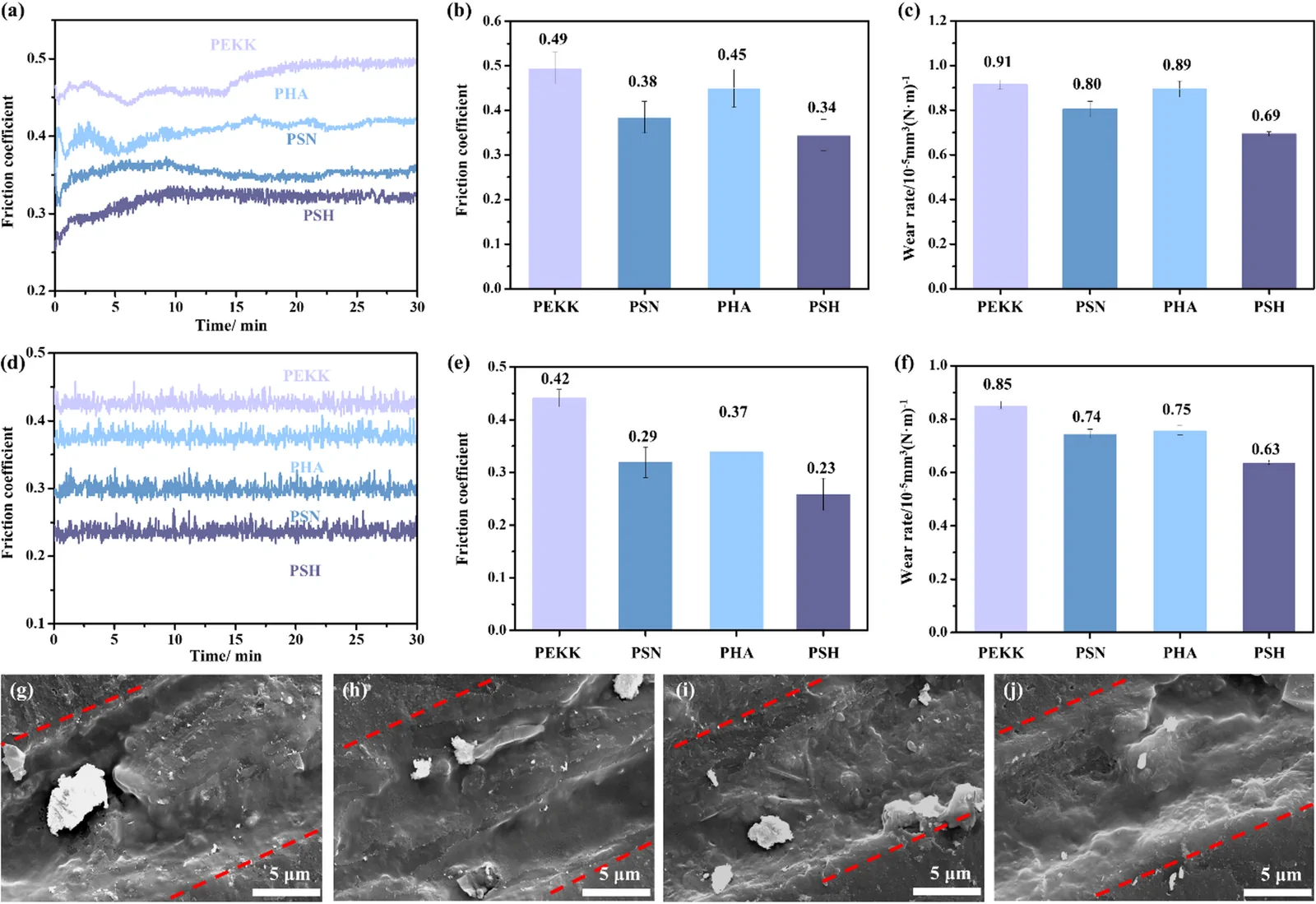
Tribological performance of PEKK-based composites. Friction coefficients and wear rates (**a-c**) under dry sliding and (**d-f**) under bovine serum lubrication; (**g-j**) Friction surface SEM images of PEKK, PSN, PHA, and PSH

Figure 3 shows the tribological performance of PEKK-based composites under dry sliding and bovine serum lubrication. As shown in Fig. 3a-c, under dry conditions, pristine PEKK exhibited a friction coefficient of 0.49 and a wear rate of 0.91 × 10^− 5^ mm^3^/N·m. For PHA, these values decreased to 0.45 and 0.89 × 10^− 5^ mm^3^/N·m. A further decrease was observed in PSN, reaching 0.38 and 0.80 × 10^− 5^ mm^3^/N·m, while the PSH composite exhibited the lowest values of 0.34 and 0.69 × 10^− 5^ mm^3^/N·m, representing reductions of 30.6% and 24.2%, respectively, compared with PEKK. Under bovine serum lubrication, all samples showed significantly lower friction and wear (Fig. 3d-f). PEKK recorded values of 0.42 and 0.85 × 10^− 5^ mm^3^/N·m. For PHA, these values decreased to 0.37 and 0.75 × 10^− 5^ mm^3^/N·m, and a further reduction was observed in PSN, reaching 0.29 and 0.74 × 10^− 5^ mm^3^/N·m. The PSH composite showed the lowest friction coefficient of 0.23 and the lowest wear rate of 0.63 × 10^− 5^ mm^3^/N·m, corresponding to 45.2% and 25.9% reductions relative to PEKK. As shown in Fig. 3g-j, the SEM images of the worn surfaces are consistent with these results. PEKK exhibited grooves accompanied by scattered abrasive debris. In general, wear debris from implant materials has been associated with local inflammatory responses, fibrous tissue formation, and impaired bone regeneration [41–43]. PHA and PSN showed grooves with some abrasive debris, while PSH presented a smoother surface with less abrasive debris, which was consistent with its lower friction coefficient and wear rate provided by the incorporation of HA and SN in PEKK. A more wear-resistant surface may help reduce local frictional damage and limit debris generation under sliding conditions, which could be beneficial for long-term interfacial stability in load-bearing applications. The improved performance of PSH is also consistent with a cooperative filler mechanism. In polymer composites, wear resistance is closely associated with load-bearing capacity, subsurface crack suppression, debris size reduction, and the stability of the interfacial transfer film [44, 45]. The addition of hard ceramic phases can reduce local plastic deformation and ploughing, while a mechanically more stable near-surface region helps maintain a more uniform sliding interface [46]. In this study, HA alone produced only a limited reduction in friction and wear, suggesting that its primary role was not tribological reinforcement. By contrast, SN nanowires likely contributed more directly to load support and resistance to groove development, which is consistent with the milder wear track morphologies observed in PSN and PSH. When combined in PSH, HA and SN appear to stabilize the near-surface microstructure through distinct but complementary physical mechanisms. Such a cooperative effect may help reduce severe abrasive damage, stabilize the sliding interface, and limit debris generation, which may explain the lower friction coefficient and wear rate observed in the PSH group.

**Fig. 4:**
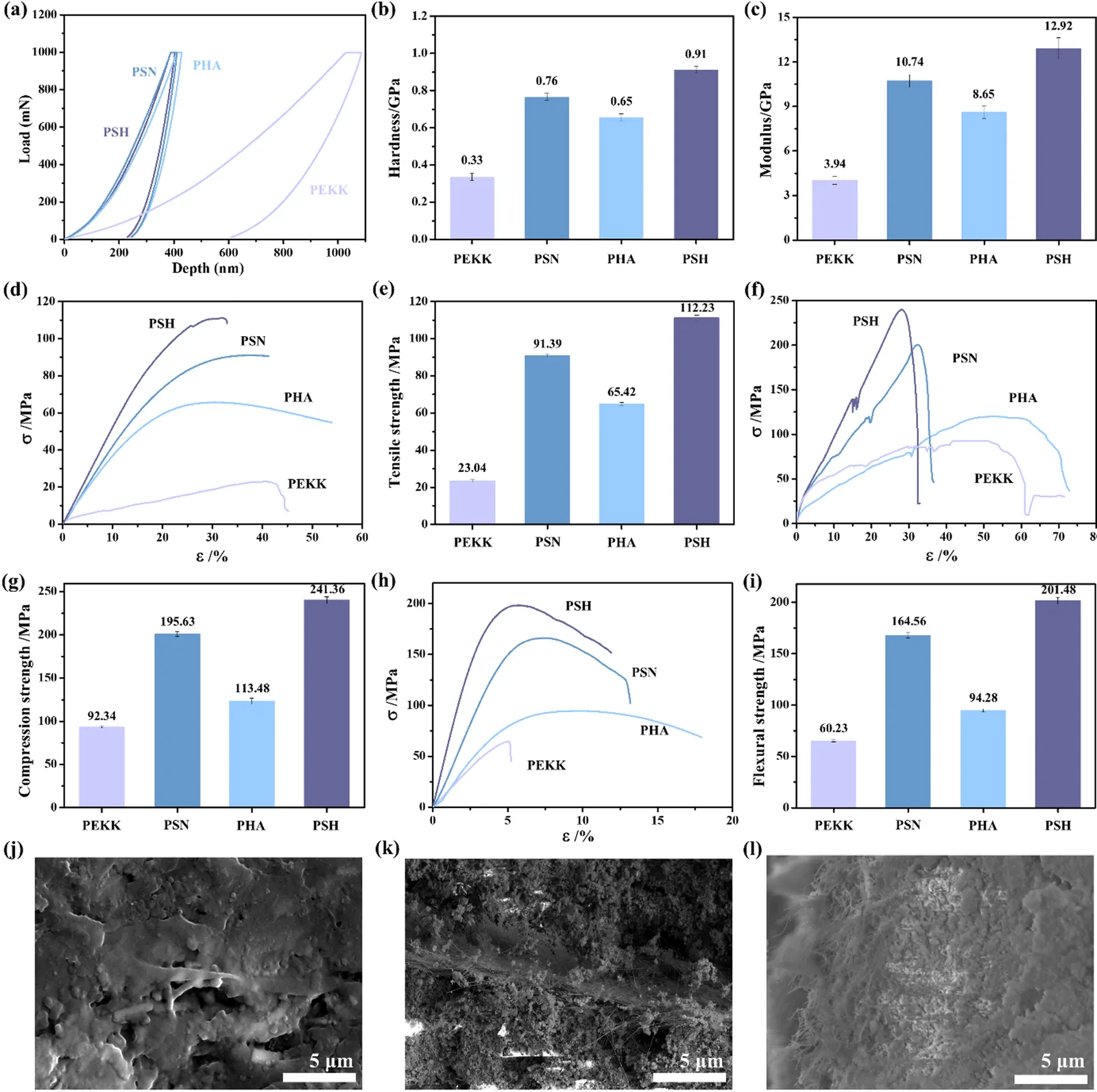
Mechanical properties and fracture morphology of PEKK composites: (**a**) Nanoindentation load–depth curves; (**b**) Hardness; (**c**) Modulus; (**d**) Tensile stress-strain curves; (**e**) Tensile strength; (**f**) Compressive stress-strain curves; (**g**) Compressive strength; (**h**) Flexural stress-strain curves; (**i**) Flexural strength. The SEM images of fracture surfaces for (**j**) PHA, (**k**) PSN, and (**l**) PSH

The elastic modulus of pure PEKK remains lower than that of cortical bone. A reduced elastic modulus can result in insufficient stress stimulation around the implant, leading to bone resorption and osteoporosis, thereby increasing healing difficulty and fracture risk [47, 48]. The nanoindentation results (Fig. 4a-c) reveal a clear enhancement in both hardness and elastic modulus after the incorporation of HA and SN nanowires into the PEKK matrix. The load-depth curves (Fig. 4a) indicate that PSH exhibits the lowest penetration depth under identical loading, indicating a higher resistance to deformation. Correspondingly, the hardness of PEKK was 0.33 GPa, which increased to 0.65 GPa for PHA, 0.76 GPa for PSN, and reached 0.91 GPa for PSH, while the elastic modulus increased from 3.94 GPa to 8.65 GPa (PHA), 10.74 GPa (PSN), and 12.92 GPa (PSH). This dual-reinforcement strategy also enables PSH to achieve an elastic modulus within the reported range of cortical bone. After implantation, a modulus closer to that of cortical bone may help improve mechanical compatibility with the surrounding bone and provide a more compatible stress-transfer environment, although functional load-bearing behavior requires further validation under physiological loading conditions [49]. In terms of tensile strength, PEKK exhibited a value of 23.04 MPa, which increased to 65.42 MPa, 91.39 MPa, and 112.23 MPa for PHA, PSN, and PSH, respectively (Fig. 4d-e). The compressive strength increased from 92.34 MPa for PEKK to 113.48 MPa, 195.63 MPa, and 241.36 MPa for PHA, PSN, and PSH, respectively (Fig. 4f-g). The flexural strength increased from approximately 60.23 MPa for PEKK to 94.28 MPa, 164.56 MPa, and 201.48 MPa for PHA, PSN, and PSH, respectively (Fig. 4h-i). These results showed that the addition of HA nanoparticles provides only limited reinforcement, while SN nanowires markedly increased the load-bearing capability of the PEKK matrix. Figure 4j-l shows the tensile fracture surface morphologies of PHA, PSN, and PSH composites. PHA (Fig. 4j) presents a relatively smooth surface, suggesting a more brittle fracture pattern and possible limited filler-matrix interaction between HA and the PEKK matrix. PSN (Fig. 4k) shows a rougher surface with evident SN nanowire pull-out and bridging, suggesting improved stress transfer and crack deflection. PSH (Fig. 4l) exhibits a compact and entangled morphology with relatively uniform distribution of SN nanowires and HA, suggesting improved filler-matrix integration and more effective stress transfer within the composite. These features support a cooperative reinforcement effect between HA and SN in improving the mechanical strength of the composite. The combination of HA and SN nanowires appears to shift the fracture morphology toward a more tortuous and energy-dissipating pattern, which is consistent with the improved mechanical properties of PEKK. This reinforcement effect can be understood from the distinct yet complementary roles of HA and SN within the PEKK matrix. In polymer nanocomposites, reinforcement efficiency is governed not only by filler stiffness, but also by filler aspect ratio, dispersion state, and interfacial stress transfer. High-aspect-ratio one-dimensional nanofillers are particularly effective in carrying applied loads and redistributing stress, while controlled interfacial debonding can promote crack deflection, frictional sliding, pull-out, and crack bridging, thereby increasing energy dissipation during fracture [50]. In the present system, HA mainly contributes stiffness enhancement and interfacial bioactivity [46], whereas SN nanowires provide a continuous reinforcing framework that bears load more efficiently and retards crack propagation [45]. This interpretation is consistent with the fracture morphologies, where PSN exhibits evident nanowire pull-out and bridging, and PSH shows a more compact fracture surface with fewer signs of catastrophic brittle separation. Notably, previous studies on HA/PEEK-type composites have shown that well-dispersed HA can improve stiffness and biointerfacial affinity but often provides limited strengthening when interfacial bonding is insufficient or particle agglomeration occurs. By contrast, the introduction of high-aspect-ratio SN nanowires in PSH likely improved stress transfer efficiency and stabilized the local fracture process, enabling HA and SN to act in a complementary manner rather than as isolated fillers. Therefore, the enhanced tensile, compressive, and flexural properties of PSH may be associated with a reinforcement mechanism involving HA-assisted stiffness increase, SN-mediated load transfer, and crack-growth resistance through bridging and pull-out mechanisms.

#### Antibacterial activity, wettability, and surface energy

**Fig. 5:**
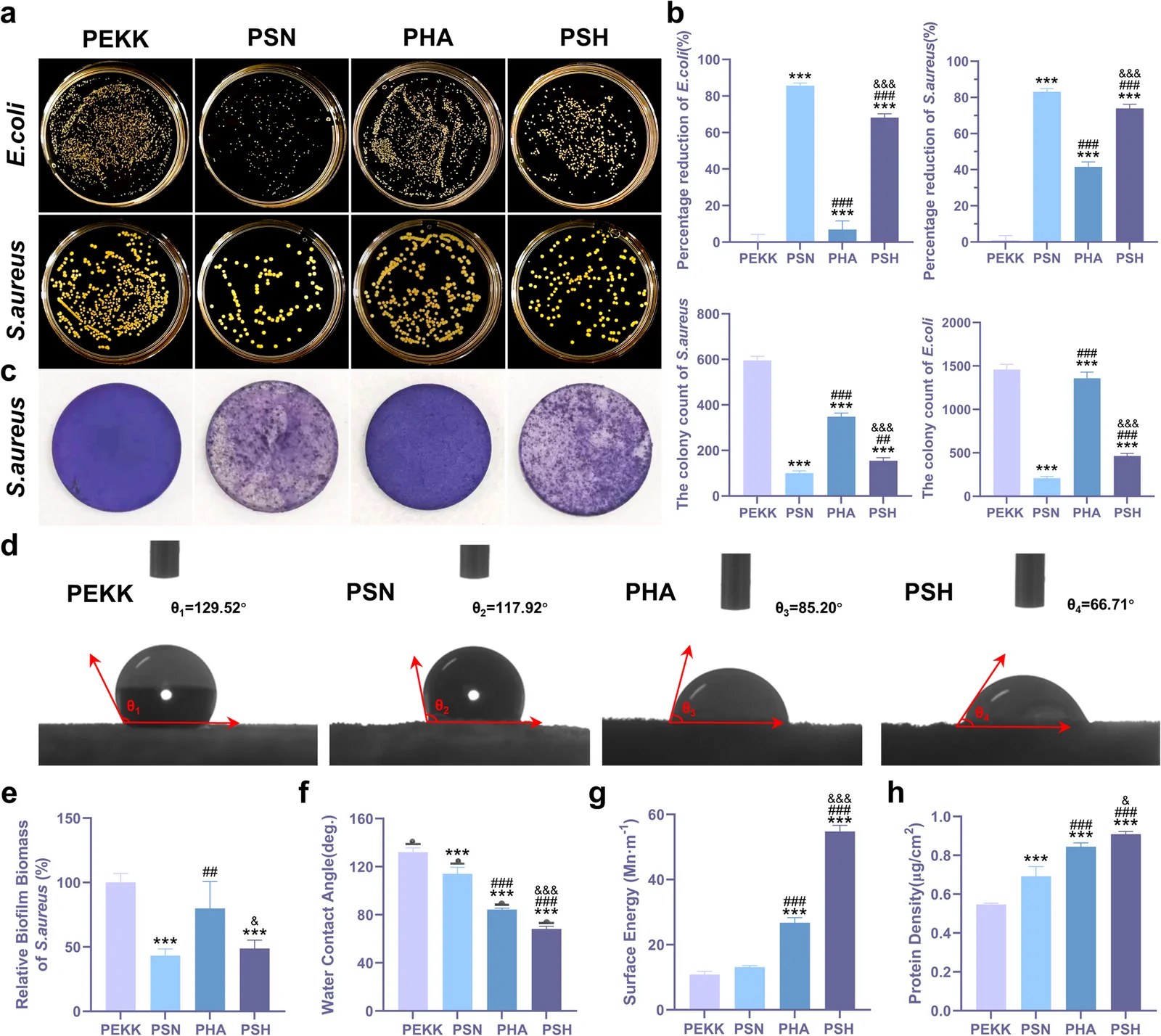
(**a**) Representative photographs of *E. coli* and *S. aureus* colonies cultured on different sample surfaces, and (**b**) the corresponding percentage reduction in bacterial viability and colony count. (**c**) Representative crystal violet-stained images of *S. aureus* biofilm formation. (**d**) Representative water droplet images on PEKK, PSN, PHA, and PSH surfaces. (**e**) Quantitative analysis of relative biofilm biomass (normalized to pristine PEKK). (**f**) Water contact angle. (**g**) Surface energy. (**h**) Protein adsorption capacity. *p < 0.05, **p < 0.01, ***p < 0.001 vs. PEKK, #*p* < 0.05, ##*p* < 0.01, ###*p* < 0.001 vs. PHA, *&p* < 0.05, &&*p* < 0.01, &&&*p* < 0.001 vs. PSH

Figure 5a presents representative colony images of *E. coli* and *S. aureus* after 24 h of culture on agar plates. Extensive bacterial growth on pristine PEKK indicated limited antibacterial activity under the tested condition. While the PHA group demonstrated only marginal inhibition, the PSN and PSH composites exhibited markedly reduced bacterial proliferation. Quantitative analysis via colony counting revealed that compared to the PEKK control, PHA achieved modest inhibition rates of 16.59% against *E. coli* and 23.88% against *S. aureus* (Fig. 5b). The PSN composite showed the highest antibacterial activity (83.76% for *E. coli* and 84.58% for *S. aureus*). PSH also showed appreciable antibacterial performance (66.81% and 75.25%, respectively), though slightly lower than that of PSN. This is likely due to the lower mass fraction of SN nanowires in the PSH ternary system [33]. The antibacterial activity conferred by SN is important for orthopedic implant materials because it may help reduce postoperative infection risk [51, 52]. To assess bacterial colonization under biofilm-forming conditions, *S. aureus*, a representative biofilm-associated pathogen in implant-related infections was cultured on the specimens in BHI broth supplemented with 1% glucose, followed by crystal violet quantification (Fig. 5c). Pristine PEKK showed the highest biofilm biomass and was used as the reference for normalization. Both SN-containing composites reduced *S. aureus* biofilm formation, with PSN exhibiting the strongest inhibition (relative biofilm biomass: 43.28 ± 5.20% of PEKK) and PSH showing a comparable but slightly weaker reduction (48.77 ± 6.51% of PEKK) (Fig. 5e). The greater inhibition observed in PSN compared with PSH is consistent with its higher SN content. These results indicate that SN nanowires contribute to the anti-biofilm activity of the composites under the tested conditions.

Based on previous studies, the antibacterial activity of SN-containing materials may involve contact-mediated bacterial membrane disturbance and local physicochemical changes at the material-bacteria interface [53, 54]. In the present study, the stronger antibacterial and biofilm-inhibitory effects of PSN than PSH are likely related to the higher SN content in PSN. However, because ion release, local pH variation, bacterial membrane integrity, and molecular-level biofilm regulatory pathways were not directly measured, the exact antibacterial mechanism of the SN-containing composites remains to be further investigated.

Surface wettability and surface energy directly influence early biological responses (Fig. 5d, f, g). Pure PEKK exhibited a highly hydrophobic character with a water contact angle of 129.52° and a minimal surface energy of 11.21 mN/m. The incorporation of SN nanowires (PSN) yielded a modest improvement, reducing the contact angle to 117.92° and increasing surface energy to 14.38 mN/m. Conversely, HA integration significantly shifted the surface toward hydrophilicity, with the PHA group recording a contact angle of 85.20° and a surface energy of 28.13 mN/m. Notably, the PSH composite showed the lowest contact angle (66.71°) and highest surface energy (56.72 mN/m) [55], which was visually corroborated by the pronounced droplet spreading (Fig. 5d).

These findings indicate that while both SN and HA individually enhance the hydrophilicity of the PEKK matrix, HA dictates the primary shift due to its highly polar surface chemistry. SN nanowires contribute in combination to surface wettability, improving surface characteristics relevant to protein adsorption and initial cell attachment (Fig. 5h), which may support subsequent osteogenic differentiation and in vivo osseointegration [56, 57].

In summary, PSN exhibited the strongest antibacterial and biofilm-inhibitory effects among the tested groups, whereas PSH retained clear antibacterial activity while showing more favorable wettability, surface energy, protein adsorption, and osteogenic potential. This balance suggests that PSH may be advantageous for applications requiring combined mechanical reinforcement, antibacterial performance, and osteogenic activity.

### In vitro cell behavior

#### Cell adhesion, proliferation and cell cycle

**Fig. 6:**
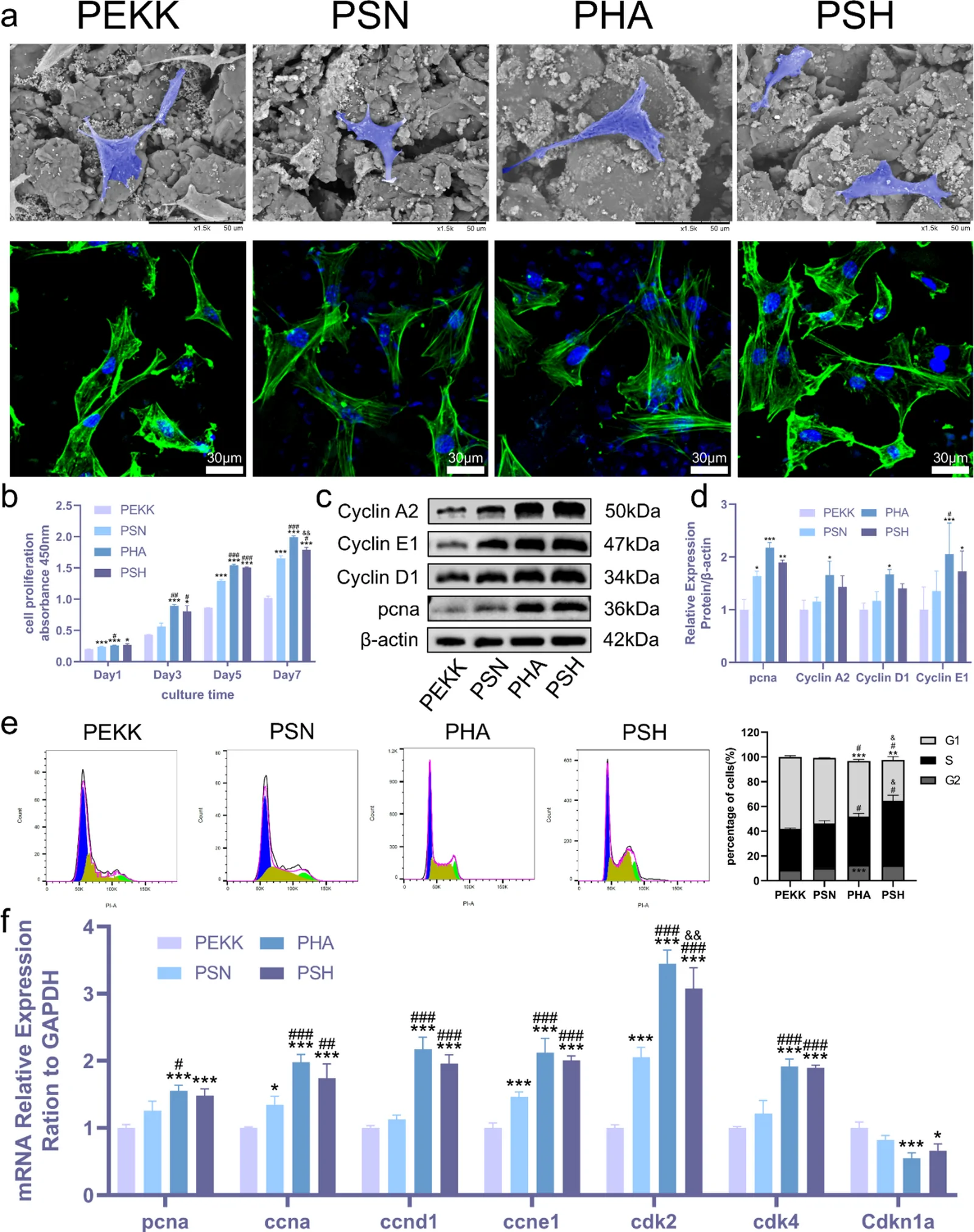
Cell adhesion, proliferation and cell proliferation-related gene and cyclin analysis. (**a**) SEM images and confocal laser scanning microscope (CLSM) observations of MC3T3-E1 cells on different samples. (Actin filaments are stained green, while the cell nuclei are stained blue). (**b**) Quantitative analysis of cell proliferation. (**c**) The protein levels of PCNA, Cyclin A2, Cyclin D1 and Cyclin E1 in MC3T3-E1 cells at day 5 were determined by western blotting. (**d**) The quantitative analysis of WB. (**e**) Cell cycle analysis by flow cytometry shows the typical cell cycle image. (**f**) The mRNA levels of cell cycle-related genes in MC3T3-E1 cells at day 5 detected by quantitative real-time PCR. *p < 0.05, **p < 0.01, ***p < 0.001 vs. PEKK, #*p* < 0.05, ##*p* < 0.01, ###*p* < 0.001 vs. PHA, &*p* < 0.05, &&*p* < 0.01, &&&*p* < 0.001 vs. PSH

Morphological evaluation by SEM and confocal laser scanning microscope (CLSM) (Fig. 6a) revealed distinct cell-material interaction patterns. Cells cultured on pristine PEKK displayed a predominantly elongated morphology and attached to the surface mainly through thin filopodia. In contrast, the introduction of SN nanowires (PSN) promoted the extension of broader pseudopodia and increased the overall spreading area. More importantly, cells seeded on the HA-incorporated surfaces (PHA and PSH) adopted a flattened polygonal morphology with well-developed lamellipodia extending across the roughened and heterogeneous surface topography, including local micro-depressions and groove-like features. Quantitative morphometric analysis further confirmed that the PHA and PSH groups exhibited significantly larger spreading areas together with relatively smaller cell volumes. Such a morphological state, characterized by extensive cytoskeletal spreading and flattening, is widely considered favorable for early osteogenic commitment and subsequent differentiation [58]. These results suggest that the HA-containing interfaces, particularly PSH, provide a more conducive microenvironment for early cell attachment and anchorage.

Cell proliferation was assessed via CCK-8 assay at Day 1, 3, 5, and 7 (Fig. 6b). All experimental groups exhibited a time-dependent increase in metabolic activity throughout the experimental period, with statistically distinct variations becoming apparent among the groups by Day 7. Subsequent experiments were conducted using cells harvested on Day 5 to further investigate these observed differences. Western blot analysis of intracellular proteins confirmed significantly elevated levels of Cyclin D1, Cyclin E1, Cyclin A2, and PCNA in the PHA and PSH groups compared with the PEKK group (Fig. 6c-d). As illustrated in Fig. 6e, cell cycle distribution analysis via flow cytometry demonstrated that HA-containing materials promoted a significant shift in cell cycle progression, characterized by an elevated proportion of cells in S phase and a corresponding reduction in G1 phase population. To investigate the molecular mechanisms underlying these phenotypic changes, we performed transcriptional profiling of key cell cycle regulators using qRT-PCR (Fig. 6f). The results revealed substantial upregulation of proliferation-associated genes (Pcna, Ccna, Ccnd1, Ccne1, Cdk2, and Cdk4) in HA-incorporated samples compared to other material groups. Concurrently, expression of Cdkn1a, a known mediator of G1 phase arrest, was significantly downregulated in these samples. Moreover, PSN also showed increased expression of these genes compared with PEKK. These results are consistent with western blotting results. In summary, the incorporation of HA and SN nanowires improved the biological response of PEKK-based surfaces, as reflected by enhanced cell cycle progression and proliferation under the tested in vitro conditions.

#### Osteogenic differentiation

**Fig. 7:**
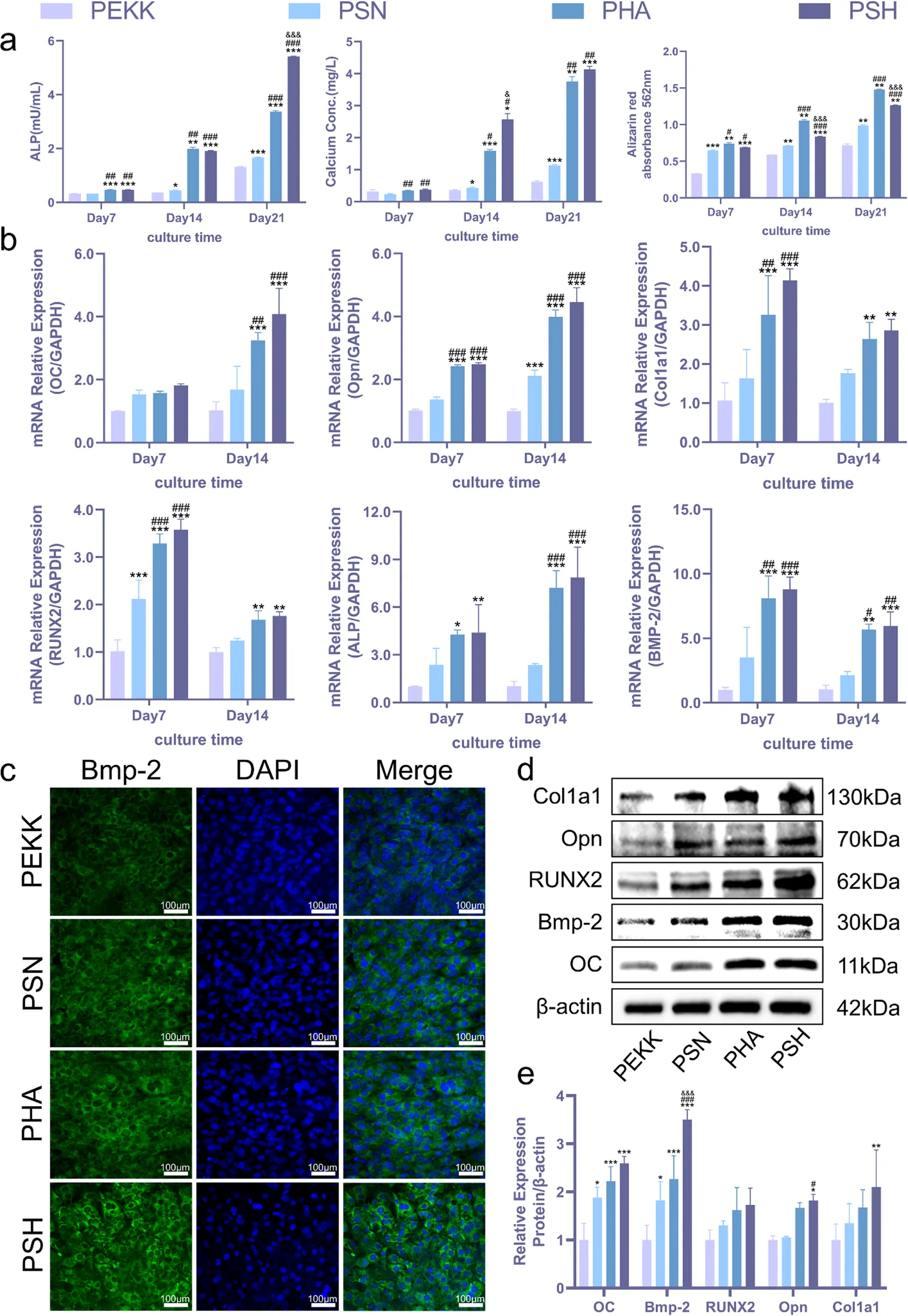
Osteogenic differentiation-related gene and protein expression and immunofluorescence staining. (**a**) Detection of alkaline phosphatase (ALP) and calcium concentration level. (**b**) qRT-PCR detection of osteogenesis-related genes. (**c**) Cells were stained for Bmp-2 (green), nuclei (blue). (**d**) The protein levels of OC, Bmp-2, RUNX2, Opn, Col1a1 in MC3T3-E1 cells on day 7 were determined by western blotting. (**e**) The quantitative analysis of WB. *p < 0.05, **p < 0.01, ***p < 0.001 vs. PEKK, #*p* < 0.05, ##*p* < 0.01, ###*p* < 0.001 vs. PHA, &*p* < 0.05, &&*p* < 0.01, &&&*p* < 0.001 vs. PSH

ALP activity, calcium concentrations and calcium nodules are key indicators for evaluating osteogenic differentiation (Figs. 7a and S4) [59]. ALP activity showed significant differences between day 7 and day 14, and significantly increased on day 21 as intergroup differences widened. The ALP activities of PHA and PSH were significantly higher than those of the other two groups at all three time points. However, the differences between the PEKK and PSN groups, as well as between the PSH and PHA groups, became significant on day 21. In contrast, on day 7, the calcium ion concentration was notably lower in the PSN group compared to the PHA and PSH groups. On day 14, the most significant inter-group difference emerged, whereas on day 21, the PEKK group exhibited a significantly lower concentration than the other three groups. Notably, the PHA and PSH groups containing HA showed significantly higher concentrations than those without HA, and the incorporation of SN also led to a significant increase in concentration. Subsequently, we examined the transcriptional levels of key osteogenic differentiation markers in MC3T3-E1 cells cultured on different materials (Fig. 7b). Quantitative analysis revealed marked upregulation of these osteogenic genes in both PHA and PSH groups compared to PEKK and PSN groups. While PSN demonstrated marginally elevated expression of certain markers relative to PEKK, these differences aligned with our earlier observations. Complementary protein-level analysis was performed using samples collected on day 7 of differentiation (Fig. 7d-e). Western blot results were consistent with increased expression of osteoblast-specific proteins in PHA and PSH groups, with PSN also showing moderately increased protein expression compared to PEKK, in agreement with the mRNA-level changes. Bmp-2, RUNX2, and Opn are mainly expressed in the early stages of osteogenesis, while OC, Col1a1, and ALP are mainly expressed in the late stages of osteogenesis. They all play important roles in the process of osteogenesis [60, 61].

To further evaluate osteogenic protein expression, we performed BMP-2 immunofluorescence staining on day 7. Bmp-2 immunofluorescence staining showed a consistent trend (Fig. 7c). Quantitative fluorescence analysis showed increased Bmp-2 expression in HA-containing groups. While the addition of SN nanowires also enhanced osteogenic protein levels, the co-incorporation of both fillers produced a stronger response than either individual modification, suggesting a cooperative contribution to osteogenic differentiation.

### In vivo animal study

#### Micro-CT analysis

**Fig. 8:**
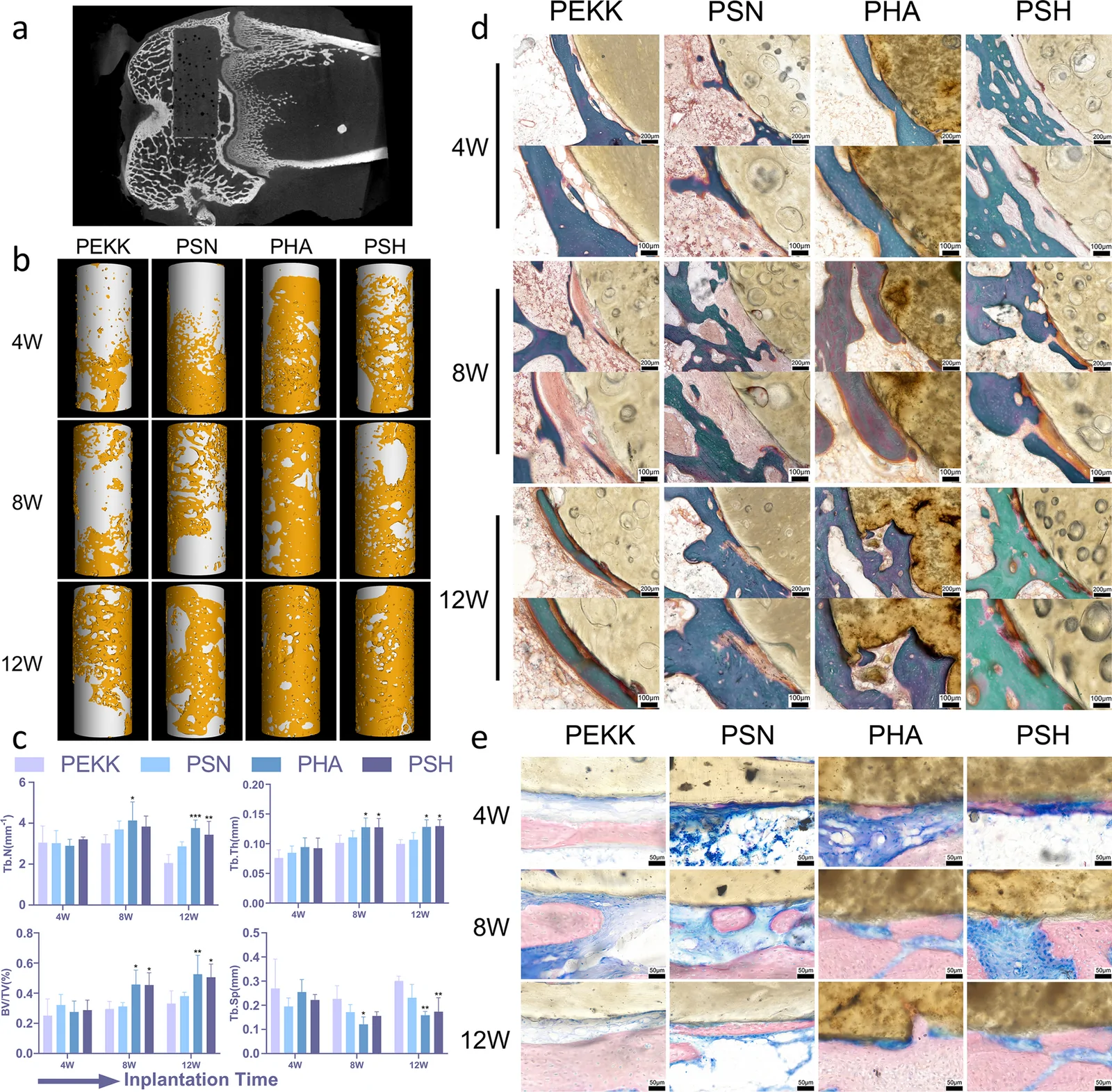
Osteogenesis analysis by Micro-CT and Histological staining. (**a**) 2D and (**b**) 3D reconstructed Micro-CT images of the implant-bone interface after implantation. (**c**) Quantitative analysis of new bone around implants at 4, 8, and 12 weeks after surgery, including bone volume/total volume (BV/TV), trabecular thickness (Tb. Th), trabecular separation (Tb. Sp) and trabecular number (Tb. N). (**d**) The observation of Goldner staining at 4, 8 and 12 weeks after the operation, respectively. Mineralized bone: blue-green; Osteoid: orange; 10X bar: 200 μm; 20X bar: 100 μm; (**e**) The observation of Methylene blue acid fuchsin staining at 4, 8 and 12 weeks after the operation, respectively. Bar: 50 μm. *p < 0.05, **p < 0.01, ***p < 0.001 vs. PEKK, #p < 0.05, ##p < 0.01, ###p < 0.001 vs. PHA, &p < 0.05, &&p < 0.01, &&&p < 0.001 vs. PSH

Micro-CT was used to evaluate the implant position within the femoral condyle and the growth of new bone within a 100 μm region surrounding the implant at weeks 4, 8 and 12. Two-dimensional (2D) reconstructed images showed that the implants were stably positioned within the femoral condyle (Fig. 8a), while 3D reconstructions (Fig. 8b) showed evident bone formation on the implant surfaces (yellow zone). However, only a small amount of newly formed bone appeared at the interface of the PEKK group. In contrast, extensive bone formation was observed at the interface in both the PHA and PSH groups, with PSH exhibiting slightly greater coverage than PHA. The PSN group also showed slightly higher bone coverage compared to PEKK. The differences became more evident by week 12. Quantitative assessment of peri-implant osteogenesis was conducted at postoperative intervals of 4, 8 and 12 weeks (Fig. 8c). The evaluation included bone volume fraction (BV/TV), trabecular thickness (Tb. Th), trabecular separation (Tb. Sp), and trabecular number (Tb. N). Both PHA and PSH showed significantly higher BV/TV, Tb. Th, and Tb. N and lower Tb. Sp than PEKK and PSN, with the differences most evident at 12 weeks. Longitudinal tracking revealed a progressive increase in bone volume at the bone-implant interface, with the PHA and PSH groups showing higher and comparable levels of new bone formation. This comparable bone mass may be related to differences in early proliferation and later osteogenic differentiation between PHA and PSH. In addition, the improved modulus and lower wear rate of PSH under in vitro tests may be beneficial for mechanical compatibility and interfacial durability. Reduced wear under the present sliding conditions may also suggest a lower tendency for wear-particle generation.

#### Histological evaluation

Figure 8d presents Goldner staining of the implanted specimens at 4, 8, and 12 weeks after surgery. New bone formation was observed at the bone-implant interface in all groups; however, only a limited amount of bone was present around the PEKK implants. A distinct gap remained between PEKK and the newly formed bone, whereas the gap around PSN was smaller. In contrast, the PHA and PSH groups exhibited much tighter contact with the surrounding bone tissue. This difference may be attributed to the relatively poor early bioactivity of PEKK, which is associated with fibrous tissue proliferation at the interface and leads to fibrous capsule formation [62–64]. Moreover, osteoid tissue was observed extending into the surface concavities of the HA-containing composites, further indicating more favorable bone-implant contact in the PHA and PSH groups.

Figure 8e shows Methylene Blue-Acid Fuchsin staining at 4, 8 and 12 weeks after surgery. In this staining, osteoblasts appear deep blue, whereas the bone matrix is stained red. The distribution of osteoblasts provides histological information on local osteogenic activity. Osteoblasts were observed around all four types of implants. More new bone tissue was present around the PHA and PSH implants, and dense osteoblasts were found within the spaces between bone trabeculae. Some new bone was also present around the PSN surface, indicating a moderate osteogenic response. In contrast, fewer osteoblasts and limited apparent bone formation were observed near the PEKK surface.

These results suggest that the combined incorporation of HA and SN improves the bioactive interface of PSH and supports peri-implant bone formation. The observed histological features are consistent with the in vitro osteogenic results and indicate closer bone-material contact in the PHA and PSH groups.

#### Fluorescent labeling of new bone formation

**Fig. 9:**
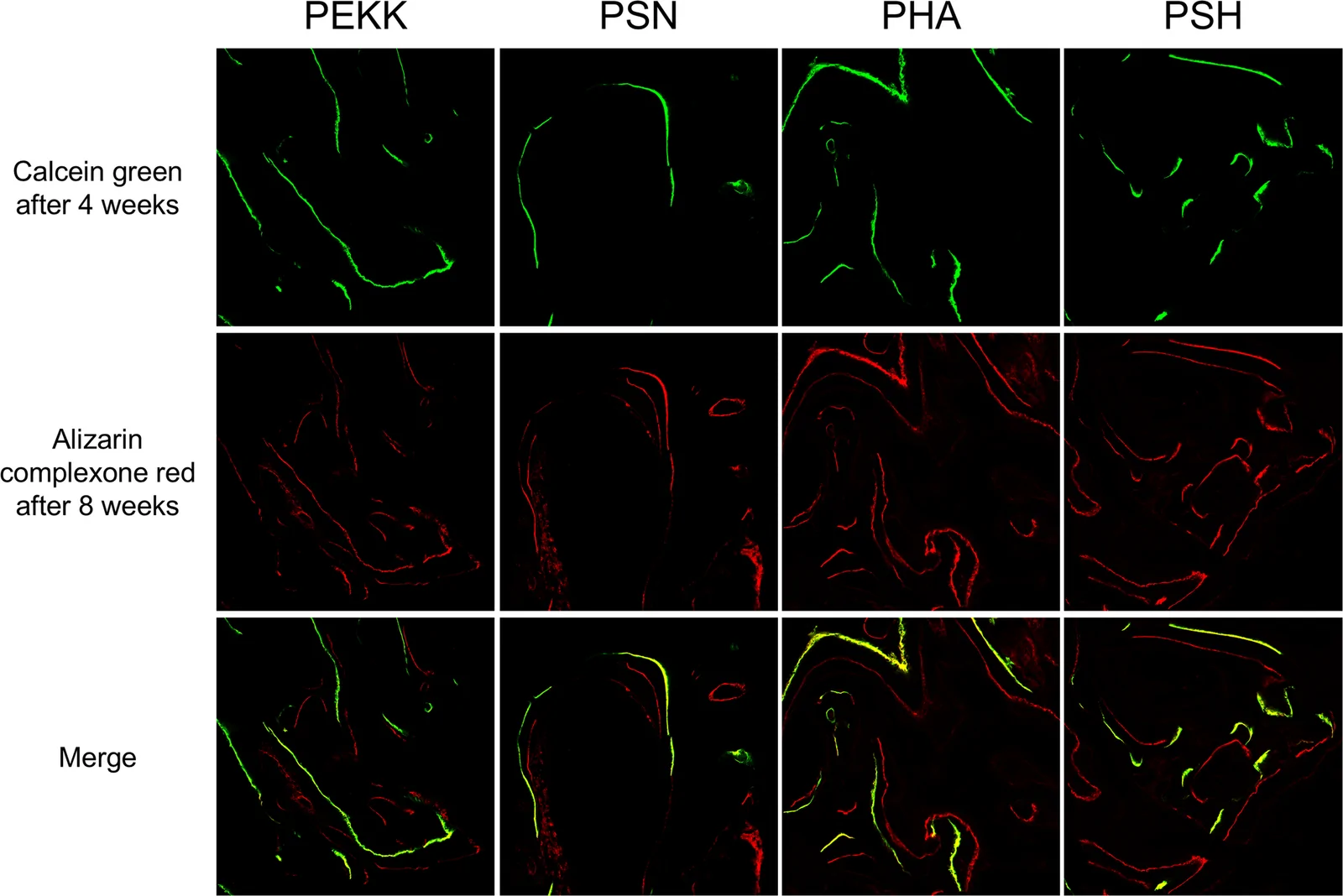
Observation of fluorescent markers of newly regenerated bone on the implants at different periods. Fluorescent labeling patterns were characterized using laser confocal microscopy after the sequential administration of calcein (week 4) and alizarin complexone (week 8)

For sequential monitoring of bone regeneration dynamics, calcein and alizarin complexone were employed as temporal markers administered at weeks 4 and 8, respectively, to distinguish newly formed bone tissue (NR bone). Sequential fluorochrome deposition generated distinct fluorescent bands, allowing for the precise spatiotemporal tracking of mineralization dynamics at the bone-implant interface [65]. The new bone formation on the surface of the material was observed using a confocal laser microscope (Fig. 9). NR bone grew on the material surface and continued to mineralize and mature progressively on the previously formed bone layer. The osteogenic ability of the materials was evaluated based on the width of NR bone labeled with different fluorochrome markers. The NR bone exhibited the greatest width on the PHA and PSH substrates, was slightly narrower on PSN, and appeared narrow and discontinuous on PEKK. At Week 4, the overall fluorescence intensity of PSN was weak, and partial discontinuity of the newly formed bone was also observed on PSH. By Week 8, the fluorescence intensity of PEKK and PSN was significantly lower than that of PHA and PSH, indicating differences in their late-stage osteogenic performance. The presence of HA was associated with enhanced new bone formation and mineralization, indicating a more favorable osteogenic response in the HA-containing materials. These results are consistent with the in vitro findings. Although early bone formation around the PSH implant appeared slightly slower than that around PHA at week 4, PSH supported osteogenic differentiation and later new bone formation within the observation period.

### Comparison with previous studies

Compared with many previously reported modified PEEK/PEKK systems, PSH was designed to improve multiple performance dimensions simultaneously rather than maximizing a single property. HA-based modifications have been widely shown to improve hydrophilicity, cell adhesion, and osteogenic activity in PEEK/PEKK platforms, particularly in porous or surface-functionalized constructs, but their contribution to mechanical reinforcement is often limited by particle agglomeration, interfacial incompatibility, or structural trade-offs associated with porous design [13, 19, 66]. In contrast, SN-containing PEEK systems have been reported to show advantages in antibacterial regulation, osteogenic stimulation, and mechanical optimization, especially in additively manufactured spinal or porous scaffold contexts [33, 67]. In PEKK-specific studies, multifunctional bioactivation has also been achieved through microporous or bioactive-particle-modified surfaces, which improved antibacterial behavior and osseointegration, although such approaches were more focused on surface bioactivity than on the integrated reinforcement of dense load-bearing composites [66, 68]. In this study, the PSH system combined HA-enabled bioactivity with SN-mediated reinforcement and resulted in concurrent improvements in mechanical, tribological, antibacterial, and osteogenic performance under the present experimental conditions.

### Limitations

Several limitations should be acknowledged in the present study. First, although the improved mechanical properties, fracture morphologies, and microstructural observations suggest improved filler–matrix integration and stress transfer in the PSH composite, the interfacial bonding among PEKK, HA, and SN nanowires was not directly quantified. More specific interfacial characterization, such as interfacial shear testing, nanoscale mechanical mapping, high-resolution cross-sectional analysis, or in situ mechanical observation, would be valuable for further clarifying the load-transfer mechanism in future studies.

Second, although PSH exhibited a lower wear rate under the present in vitro sliding conditions, the biological effects of composite wear debris were not directly evaluated. In particular, the size distribution, chemical composition, and dose-dependent biological responses of wear particles were not analyzed, and macrophage-mediated inflammatory responses were not assessed. Therefore, the current wear data should be interpreted as evidence of improved tribological performance under defined in vitro conditions, rather than as direct proof of long-term wear-debris-related biosafety.

Third, the present study observed antibacterial activity against *E. coli* and *S. aureus*, and the *S. aureus* biofilm assay further showed that SN-containing composites reduced biofilm formation under an in vitro biofilm-forming condition. However, this assay characterized biofilm formation under static in vitro conditions rather than the disruption or eradication of pre-formed mature biofilms in dynamic environments. In addition, ion release, local pH variation, bacterial membrane integrity, and molecular-level biofilm regulatory pathways were not directly measured. Therefore, the antibacterial and biofilm-inhibitory mechanisms of the SN-containing composites require further investigation.

Fourth, although SN nanowires contributed to mechanical reinforcement and antibacterial performance, the long-term structural stability and possible degradation behavior of SN nanowires within the PEKK matrix were not investigated. These aspects are important for evaluating the durability and long-term biological performance of the composite under clinically relevant conditions.

Finally, the in vivo study was conducted in a rabbit femoral condyle model with a relatively limited observation period and sample size. This model is useful for evaluating peri-implant bone formation but may not fully reproduce the complex mechanical loading, long-term bone remodeling, and wear environment of load-bearing orthopedic implants in clinical practice. Future studies should further investigate long-term interfacial stability, wear-debris-related biosafety, antibacterial mechanisms, and functional performance under more physiologically relevant loading and implantation conditions.

## Conclusion

In this study, a dual-reinforced PEKK-based composite was developed by incorporating hydroxyapatite (HA) particles and silicon nitride (SN) nanowires into the PEKK matrix. The ternary PEKK/HA/SN composite (PSH) exhibited improved mechanical strength, tribological performance, antibacterial activity, and osteogenic response compared with pristine PEKK. PSH achieved tensile, compressive, and flexural strengths of 112.23, 241.36, and 201.48 MPa, respectively, and showed the lowest friction coefficient and wear rate under both dry and bovine-serum-lubricated conditions among the tested groups. In addition, PSH retained antibacterial activity against E. coli and S. aureus and reduced S. aureus biofilm formation under an in vitro biofilm-forming condition. The improved wettability, surface energy, and protein adsorption of PSH were accompanied by enhanced osteoblast proliferation and osteogenic differentiation in vitro. In vivo, PHA and PSH supported improved peri-implant bone formation in a rabbit femoral condyle model, with comparable bone mass and density at week 12. Overall, these findings suggest that the combined incorporation of HA and SN nanowires is a feasible strategy for improving the mechanical, tribological, antibacterial, and osteogenic performance of PEKK. Further work, including direct interfacial characterization, wear-debris biological evaluation, and longer-term in vivo assessment under physiologically relevant loading, is needed to clarify the underlying mechanisms and translational relevance of the present findings.

## Supplementary Information

Below is the link to the electronic supplementary material.

Supplementary Material 1.

Supplementary Material 2.

## Data Availability

This paper presents all data required for the evaluation of its conclusions. Requests for further information related to this work can be directed to the corresponding author.

## References

[1] Zol SM, Alauddin MS, Said Z, Mohd Ghazali MI, Hao-Ern L, Mohd Farid DA, et al. Description of Poly(aryl-ether-ketone) Materials (PAEKs), Polyetheretherketone (PEEK) and Polyetherketoneketone (PEKK) for Application as a Dental Material. Mater Sci Rev Polym. 2023;15:2170. 10.3390/polym15092170. PMC10180673 37177316

[2] Zhu H, Liu Z, Lu M, Han X, Zhang S. Exploring PEKK: properties, applications and promise of personalized medicine. J Mater Chem B. 2025;13:7934–52. 10.1039/D5TB00596E. 40491219

[3] Kersten RFMR, Van Gaalen SM, De Gast A, Öner FC. Polyetheretherketone (PEEK) cages in cervical applications: a systematic review. Spine J. 2015;15:1446–60. 10.1016/j.spinee.2013.08.030. 24374100

[4] Gu P, Wen Z, Bao L, Wang Y, Ouyang P, Lu T, et al. New synthetic PEKK/bioceramic hybrids and their surface sulfonation counterparts have increased cellular osteogenic capacity and promoted osseointegration. Mater Des. 2022;224:111283. 10.1016/j.matdes.2022.111283.

[5] Cunningham BW, Brooks DM, Rolle NP, Weiner DA, Wang W. An investigational time course study of titanium plasma spray on osseointegration of PEEK and titanium implants: an in vivo ovine model. Spine J. 2024;24:721–9. 10.1016/j.spinee.2023.10.005. 37875243

[6] Jiang P, Zhang Y, Hu R, Shi B, Zhang L, Huang Q, et al. Advanced surface engineering of titanium materials for biomedical applications: From static modification to dynamic responsive regulation. Bioact Mater. 2023;27:15–57. 10.1016/j.bioactmat.2023.03.006. 37035422 PMC10074421

[7] Kurtz SM, Devine JN. PEEK biomaterials in trauma, orthopedic, and spinal implants. Biomaterials. 2007;28:4845–69. 10.1016/j.biomaterials.2007.07.013. 17686513 PMC2040108

[8] Dondani JR, Iyer J, Tran SD. Surface Treatments of PEEK for Osseointegration to Bone. Biomolecules. 2023;13:464. 10.3390/biom13030464. 36979399 PMC10046336

[9] He M, Huang Y, Xu H, Feng G, Liu L, Li Y, et al. Modification of polyetheretherketone implants: From enhancing bone integration to enabling multi-modal therapeutics. Acta Biomater. 2021;129:18–32. 10.1016/j.actbio.2021.05.009. 34020056

[10] Yang X, Wang Q, Zhang Y, He H, Xiong S, Chen P, et al. A dual-functional PEEK implant coating for anti-bacterial and accelerated osseointegration. Colloids Surf B Biointerfaces. 2023;224:113196. 10.1016/j.colsurfb.2023.113196. 36764204

[11] Wang Z, Yu Z, Wang Z, Li S, Song L, Xu T, et al. Surface-activated 3D-printed PEEK implant enhances anti-infection and osteogenesis. Compos Part B Eng. 2024;273:111258. 10.1016/j.compositesb.2024.111258.

[12] Yuan B, Zhang Y, Zhao R, Lin H, Yang X, Zhu X, et al. A unique biomimetic modification endows polyetherketoneketone scaffold with osteoinductivity by activating cAMP/PKA signaling pathway. Sci Adv. 2022;8:eabq7116. 10.1126/sciadv.abq7116. 36197987 PMC9534509

[13] Liu H, Liu T, Yin Z, Liu X, Tan Y, Zhao Y, et al. Bio-functional hydroxyapatite-coated 3D porous polyetherketoneketone scaffold for enhanced osteogenesis and osteointegration in orthopedic applications. Regen Biomater. 2024;11:rbae023. 10.1093/rb/rbae023. 38559647 PMC10980557

[14] Lommen J, Schorn L, Sproll C, Kübler NR, Nicolini LF, Merfort R, et al. Mechanical Fatigue Performance of Patient-Specific Polymer Plates in Oncologic Mandible Reconstruction. J Clin Med. 2022;11:3308. 10.3390/jcm11123308. 35743379 PMC9224531

[15] Katzenbach A, Dörsam I, Stark H, Bourauel C, Keilig L. Fatigue behaviour of dental crowns made from a novel high-performance polymer PEKK. Clin Oral Investig. 2021;25:4895–905. 10.1007/s00784-021-03797-9. 33506428 PMC8342378

[16] Wu F, Chen H, Fang H, Wu Y, Fang Y, Lin H. Ultra-low wear performance of PEKK composites synergistically reinforced with short carbon fibers and PTFE. Tribol Int. 2026;216:111520. 10.1016/j.triboint.2025.111520.

[17] Wang Z, Xiang Q, Tan X, Zhang Y, Zhu H, Pu J, et al. Functionalized Cortical Bone-Inspired Composites Adapt to the Mechanical and Biological Properties of the Edentulous Area to Resist Fretting Wear. Adv Sci. 2023;10:2207255. 10.1002/advs.202207255. PMC10104646 36775879

[18] Liu X, Ouyang L, Chen L, Qiao Y, Ma X, Xu G, et al. Hydroxyapatite composited PEEK with 3D porous surface enhances osteoblast differentiation through mediating NO by macrophage. Regen Biomater. 2022;9:rbab076. 10.1093/rb/rbab076. 35480864 PMC9039504

[19] Zheng J, Zhao H, Dong E, Kang J, Liu C, Sun C, et al. Additively-manufactured PEEK/HA porous scaffolds with highly-controllable mechanical properties and excellent biocompatibility. Mater Sci Eng C. 2021;128:112333. 10.1016/j.msec.2021.112333. 34474884

[20] Kang J, Zheng J, Hui Y, Li D. Mechanical Properties of 3D-Printed PEEK/HA Composite Filaments. Polymers. 2022;14:4293. 10.3390/polym14204293. 36297871 PMC9608599

[21] Rodzeń K, Sharma PK, McIlhagger A, Mokhtari M, Dave F, Tormey D, et al. The Direct 3D Printing of Functional PEEK/Hydroxyapatite Composites via a Fused Filament Fabrication Approach. Polymers. 2021;13:545. 10.3390/polym13040545. 33673299 PMC7917676

[22] Liu Y, Zhang L, Nie H, Sheng H, Li H. Balanced Mechanical and Biotribological Properties of Polymer Composites Reinforced by a 3D Interlocked Si_3_N_4_ Nanowire Membrane. ACS Appl Mater Interfaces. 2022;14:56203–12. 10.1021/acsami.2c19535. 36484566

[23] Zhang L, Zhang Y, Zhu F, Zhao Z, Yang Y, Sheng H, et al. SiC Nanowire–Si_3_N_4_ Nanobelt Interlocking Interfacial Enhancement of Carbon Fiber Composites with Boosting Mechanical and Frictional Properties. ACS Appl Mater Interfaces. 2021;13:20746–53. 10.1021/acsami.1c04682. 33896188

[24] Diniță A, Ripeanu RG, Ilincă CN, Cursaru D, Matei D, Naim RI, et al. Advancements in Fiber-Reinforced Polymer Composites: A Comprehensive Analysis. Polymers. 2023;16:2. 10.3390/polym16010002. 38201669 PMC10780509

[25] Zeng S, Li H, Hu P, Li Z, Wang Z, Wang J, et al. Additive manufacturing of silicon nitride fiber-reinforced polyetheretherketone composites with enhanced mechanical strength and multifunctional bioactivity for load-bearing bone defect repair. Biofabrication. 2025;17:035014. 10.1088/1758-5090/add9d3. 40378856

[26] Sun L, Zhang L, Wang J, Liu Y, Guo Y. Fabrication of novel multilayer core-shell structured nanofibers network reinforced carbon matrix composites for bone tissue engineering. Mater Lett. 2023;333:133634. 10.1016/j.matlet.2022.133634.

[27] Du S, Li F, Zhang J, Chen Z, Zhang S, Zhao S, et al. Effects of sintering additives and sintering methods on the mechanical, antimicrobial and optical properties of Si3N4 bioceramics. J Mech Behav Biomed Mater. 2024;154:106529. 10.1016/j.jmbbm.2024.106529. 38552335

[28] He J, Liu Y, Zeng X, Tong Y, Liu R, Wang K, et al. Silicon Nitride Bioceramics Sintered by Microwave Exhibit Excellent Mechanical Properties, Cytocompatibility In Vitro, and Anti-Bacterial Properties. J Funct Biomater. 2023;14:552. 10.3390/jfb14110552. 37998121 PMC10671902

[29] Webster TJ, Patel AA, Rahaman MN, Sonny Bal B. Anti-infective and osteointegration properties of silicon nitride, poly(ether ether ketone), and titanium implants. Acta Biomater. 2012;8:4447–54. 10.1016/j.actbio.2012.07.038. 22863905

[30] Tani A, Tsubouchi H, Ma L, Taniguchi Y, Kobayashi Y, Nakai M, et al. Effect of Silicon Nitride Coating on Titanium Surface: Biocompatibility and Antibacterial Properties. Int J Mol Sci. 2024;25:9148. 10.3390/ijms25179148. 39273096 PMC11394916

[31] Wu H, Liu T, Xu Z, Qian J, Shen X, Li Y, et al. Enhanced bacteriostatic activity, osteogenesis and osseointegration of silicon nitride/polyetherketoneketone composites with femtosecond laser induced micro/nano structural surface. Appl Mater Today. 2020;18:100523. 10.1016/j.apmt.2019.100523.

[32] Huang Z, Zhou H, Yuan F, Wu J, Yuan S, Cai K, et al. Investigation on the Osteogenic and Antibacterial Properties of Silicon Nitride-Coated Titanium Dental Implants. ACS Biomater Sci Eng. 2024;10:4059–72. 10.1021/acsbiomaterials.4c00427. 38748565

[33] Basgul C, DeSantis P, Derr T, Hickok NJ, Bock RM, Kurtz SM. Exploring the mechanical strength, antimicrobial performance, and bioactivity of 3D-printed silicon nitride-PEEK composites in cervical spinal cages. Int J Bioprinting. 2024;10:2124. 10.36922/ijb.2124. PMC12406975 40909184

[34] Hu G, Zhu Y, Xu F, Ye J, Guan J, Jiang Y, et al. Comparison of surface properties, cell behaviors, bone regeneration and osseointegration between nano tantalum/PEEK composite and nano silicon nitride/PEEK composite. J Biomater Sci Polym Ed. 2022;33:35–56. 10.1080/09205063.2021.1974812. 34464239

[35] Pezzotti G, Oba N, Zhu W, Marin E, Rondinella A, Boschetto F, et al. Human osteoblasts grow transitional Si/N apatite in quickly osteointegrated Si3N4 cervical insert. Acta Biomater. 2017;64:411–20. 10.1016/j.actbio.2017.09.038. 28963015

[36] Arquier R, Iliopoulos I, Régnier G, Miquelard-Garnier G. Consolidation of continuous-carbon-fiber-reinforced PAEK composites: a review. Mater Today Commun. 2022;32:104036. 10.1016/j.mtcomm.2022.104036.

[37] Yildirim C, Tabrizi IE, Al-Nadhari A, Topal S, Beylergil B, Yildiz M. Characterizing damage evolution of CF/PEKK composites under tensile loading through multi-instrument structural health monitoring techniques. Compos Part Appl Sci Manuf. 2023;175:107817. 10.1016/j.compositesa.2023.107817.

[38] Pérez-Martín H, Mackenzie P, Baidak A, Ó Brádaigh CM, Ray D. Crystallinity studies of PEKK and carbon fibre/PEKK composites: A review. Compos Part B Eng. 2021;223:109127. 10.1016/j.compositesb.2021.109127.

[39] Pérez-Martín H, Mackenzie P, Baidak A, Ó Brádaigh CM, Ray D. Crystallisation behaviour and morphological studies of PEKK and carbon fibre/PEKK composites. Compos Part Appl Sci Manuf. 2022;159:106992. 10.1016/j.compositesa.2022.106992.

[40] Pérez-Martín H, Mackenzie P, Baidak A, Ó Brádaigh CM, Ray D. Microstructural and micromechanical property characterisation of CF/PEKK composites using nanoindentation. Mater Des. 2023;234:112359. 10.1016/j.matdes.2023.112359.

[41] Wu T, Zhang X, Chen K, Chen Q, Yu Z, Feng C, et al. The antibacterial and wear-resistant nano-ZnO/PEEK composites were constructed by a simple two-step method. J Mech Behav Biomed Mater. 2022;126:104986. 10.1016/j.jmbbm.2021.104986. 34856483

[42] Patnaik L, Kumar S, Gajjar J, Deepak A, Naidana J, Venkatesh VSS, et al. Carbon-fibre-reinforced-PEEK and silicon doped amorphous carbon as a potential tribopair for implant application. Adv Mater Process Technol. 2024;10:2761–79. 10.1080/2374068X.2023.2184337.

[43] Fleischhacker E, Sprecher C, Milz S, Saller M, Wirz R, Zboray R, et al. Inflammatory tissue response in human soft tissue is caused by a higher particle load near carbon fiber-reinforced PEEK compared to titanium plates. Acta Biomater. 2024;180:128–39. 10.1016/j.actbio.2024.04.023. 38636789

[44] Panin SV, Alexenko VO, Buslovich DG. High Performance Polymer Composites: A Role of Transfer Films in Ensuring Tribological Properties—A Review. Polymers. 2022;14:975. 10.3390/polym14050975. 35267795 PMC8912496

[45] Chan JX, Wong JF, Petrů M, Hassan A, Nirmal U, Othman N, et al. Effect of Nanofillers on Tribological Properties of Polymer Nanocomposites: A Review on Recent Development. Polymers. 2021;13:2867. 10.3390/polym13172867. 34502906 PMC8433795

[46] Wang L, Weng L, Song S, Zhang Z, Tian S, Ma R. Characterization of polyetheretherketone–hydroxyapatite nanocomposite materials. Mater Sci Eng A. 2011;528:3689–96. 10.1016/j.msea.2011.01.064.

[47] Naghavi SA, Lin C, Sun C, Tamaddon M, Basiouny M, Garcia-Souto P, et al. Stress Shielding and Bone Resorption of Press-Fit Polyether–Ether–Ketone (PEEK) Hip Prosthesis: A Sawbone Model Study. Polymers. 2022;14:4600. 10.3390/polym14214600. 36365594 PMC9657056

[48] Milone D, Spataro M. Biomechanical Evaluation of PEEK and PLA Composite Femoral Implants for Stress Shielding Reduction: A Finite Element Simulation Study. Prosthesis. 2025;7:44. 10.3390/prosthesis7030044.

[49] Sun Q, Hou Y, Chu Z, Wei Q. Soft overcomes the hard: Flexible materials adapt to cell adhesion to promote cell mechanotransduction. Bioact Mater Elsevier BV. 2022;10:397–404. 10.1016/j.bioactmat.2021.08.026. PMC8636665 34901555

[50] Fu S, Sun Z, Huang P, Li Y, Hu N. Some basic aspects of polymer nanocomposites: A critical review. Nano Mater Sci. 2019;1:2–30. 10.1016/j.nanoms.2019.02.006.

[51] Masters EA, Ricciardi BF, Bentley KLDM, Moriarty TF, Schwarz EM, Muthukrishnan G. Skeletal infections: microbial pathogenesis, immunity and clinical management. Nat Rev Microbiol. 2022;20:385–400. 10.1038/s41579-022-00686-0. 35169289 PMC8852989

[52] Wang L, He H, Yang X, Zhang Y, Xiong S, Wang C, et al. Bimetallic ions regulated PEEK of bone implantation for antibacterial and osteogenic activities. Mater Today Adv. 2021;12:100162. 10.1016/j.mtadv.2021.100162.

[53] Du X, Lee SS, Blugan G, Ferguson SJ. Silicon Nitride as a Biomedical Material: An Overview. Int J Mol Sci. 2022;23:6551. 10.3390/ijms23126551. 35742996 PMC9224221

[54] Boschetto F, Rondinella A, Marin E. Biological Activity of Silicon Nitride Ceramics: A Critical Review. Materials. 2024;17:5548. 10.3390/ma17225548. 39597372 PMC11595669

[55] Chrzanowski W, Lee JH, Kondyurin A, Lord MS, Jang J, Kim H, et al. Nano-Bio‐Chemical Braille for Cells: The Regulation of Stem Cell Responses using Bi‐Functional Surfaces. Adv Funct Mater. 2015;25:193–205. 10.1002/adfm.201401696.

[56] Liu X, Chu PK, Ding C. Surface nano-functionalization of biomaterials. Mater Sci Eng R Rep. 2010;70:275–302. 10.1016/j.mser.2010.06.013.

[57] Chen T, Jinno Y, Atsuta I, Tsuchiya A, Obinata S, Iimori R, et al. Synergistic Effect of Nano Strontium Titanate Coating and Ultraviolet C Photofunctionalization on Osteogenic Performance and Soft Tissue Sealing of poly(ether–ether–ketone). ACS Biomater Sci Eng. 2024;10:825–37. 10.1021/acsbiomaterials.3c01684. 38267012 PMC10866145

[58] Guo M, Pegoraro AF, Mao A, Zhou EH, Arany PR, Han Y, et al. Cell volume change through water efflux impacts cell stiffness and stem cell fate. Proc Natl Acad Sci. 2017;114(41):E8618–27. 10.1073/pnas.1705179114. 28973866 PMC5642688

[59] Chen J, Zhou H, Fan Y, Gao G, Ying Y, Li J. 3D printing for bone repair: Coupling infection therapy and defect regeneration. Chem Eng J. 2023;471:144537. 10.1016/j.cej.2023.144537.

[60] Fang Y, Wang Q, Yang Z, Yang W, Wang L, Ma J, et al. An efficient approach to endow TiNbTaZr implant with osteogenic differentiation and antibacterial activity in vitro. Mater Des. 2022;221:110987. 10.1016/j.matdes.2022.110987.

[61] Pativada T, Kim MH, Lee J-H, Hong SS, Choi CW, Choi Y-H, et al. Benzylideneacetone Derivatives Inhibit Osteoclastogenesis and Activate Osteoblastogenesis Independently Based on Specific Structure–Activity Relationship. J Med Chem. 2019;62:6063–82. 10.1021/acs.jmedchem.9b00270. 31257875

[62] Al Maruf DSA, Ren J, Cheng K, Xin H, Lewin W, Pickering E, et al. Evaluation of osseointegration of plasma treated polyaryletherketone maxillofacial implants. Sci Rep. 2025;15:1895. 10.1038/s41598-024-80335-z. 39805882 PMC11731023

[63] Zhang C, Xie E, Zhong Z, Wang F, Xie S, Huang S, et al. Incorporation of tantalum into PEEK and grafting of berbamine facilitate osteoblastogenesis for enhancing osseointegration and inhibit osteoclastogenesis for preventing aseptic loosening. Compos Part B Eng. 2025;296:112242. 10.1016/j.compositesb.2025.112242.

[64] Feng P, Wu P, Gao C, Yang Y, Guo W, Yang W, et al. A Multimaterial Scaffold With Tunable Properties: Toward Bone Tissue Repair. Adv Sci. 2018;5:1700817. 10.1002/advs.201700817. PMC6033191 29984132

[65] Ducy P, Schinke T, Karsenty G. The Osteoblast: A Sophisticated Fibroblast under Central Surveillance. Science. 2000;289:1501–4. 10.1126/science.289.5484.1501. 10968779

[66] Hu X, Mei S, Wang F, Tang S, Xie D, Ding C, et al. A microporous surface containing Si3N4/Ta microparticles of PEKK exhibits both antibacterial and osteogenic activity for inducing cellular response and improving osseointegration. Bioact Mater. 2021;6:3136–49. 10.1016/j.bioactmat.2021.02.027. 33778194 PMC7960946

[67] Du X, Ronayne S, Lee SS, Hendry J, Hoxworth D, Bock R, et al. 3D-Printed PEEK/Silicon Nitride Scaffolds with a Triply Periodic Minimal Surface Structure for Spinal Fusion Implants. ACS Appl Bio Mater. 2023;6:3319–29. 10.1021/acsabm.3c00383. 37561906 PMC10445264

[68] Zheng Z, Liu P, Zhang X, Xin J, Wang Y, Zou X, et al. Strategies to improve bioactive and antibacterial properties of polyetheretherketone (PEEK) for use as orthopedic implants. Mater Today Bio. 2022;16:100402. 10.1016/j.mtbio.2022.100402. 36105676 PMC9466655

