# Supplementary material for: Dual reinforcement of PEKK composites with hydroxyapatite and silicon nitride nanowires for enhanced mechanical, tribological, antibacterial, and osteogenic performance
Source: J Nanobiotechnology. 2026 Jun 26;24:853. doi: 10.1186/s12951-026-04708-4 (PMC13560440; doi:10.1186/s12951-026-04708-4)
Supplement: Supplementary file 1 [file 12951_2026_4708_MOESM1_ESM.docx]

Fig. 6c-Cyclin A2 (the left group)

Fig. 6c-Cyclin E1 (the right group)

Fig. 6c-Cyclin D1 (the left group)

Fig. 6c-pcna (the middle group)

Fig. 6c-β-actin

Fig. 7d-Col1a1

Fig. 7d-Opn (the left group)

Fig. 7d-RUNX2 (the right group)

Fig. 7d-Bmp-2

Fig. 7d-OC

Fig. 7d-β-actin
