# Supplementary material for: Dual reinforcement of PEKK composites with hydroxyapatite and silicon nitride nanowires for enhanced mechanical, tribological, antibacterial, and osteogenic performance
Source: J Nanobiotechnology. 2026 Jun 26;24:853. doi: 10.1186/s12951-026-04708-4 (PMC13560440; doi:10.1186/s12951-026-04708-4)
Supplement: Supplementary file 2 [file 12951_2026_4708_MOESM2_ESM.docx]

**Support Information**

**Dual Reinforcement of PEKK Composites with Hydroxyapatite and Silicon Nitride Nanowires for Enhanced Mechanical, Tribological, Antibacterial, and Osteogenic Performance**

*Yuewen Xue^a1^ Lina Sun^b1^, Qinghua Tan^a^, Tongtong Cui^c^, Wanzhu Hao^d^, Xia Luo^e^, Ningyi Xian^f^, Xijing He^a, g^*^[1]^**, Leilei Zhang^b^*^[2]^**, Ting Zhang^a^*^[3]^*

a Department of Orthopedics, The Second Affiliated Hospital of Xi’an Jiaotong University, Xi’an 710000, P. R. China

b State Key Laboratory of Solidification Processing, Shaanxi Key Laboratory of Fiber Reinforced Light Composite Materials, Northwestern Polytechnical University, Xi’an 710072, P. R. China

c Institute for Technical Chemistry and Polymer Chemistry (ITCP), Karlsruhe Institute of Technology (KIT), Engesserstraße 18, 76131 Karlsruhe, Germany

d Department of Rehabilitation, Beijing Rehabilitation Hospital of Capital Medical University, Beijing, 100043, P. R. China

e Department of Medical Imaging, The First Affiliated Hospital of Xi’an Jiaotong University, Xi’an 710000, P. R. China

f Department of Dermatology, The First Affiliated Hospital of Xi’an Jiaotong University, Xi’an 710000, P. R. China

g Department of Orthopedics, Xi’an International Medical Center Hospital, Xi’an 710000, P. R. China

**Fig. S1.** Schematic diagram of the SLGS growth model of SN.

The growth of SN preforms is proposed to follow a solid-liquid-gas-solid (SLGS) mechanism. The precursor PSN is first cross-linked to cure at 200-300℃. The eutectic temperature of Si-C-N ceramics is about 1300 ℃, so when the heating temperature is higher than the eutectic temperature, the liquid of Si-C-N will be formed. Next, by constantly consuming Si-C-N ceramics and absorbing nitrogen elements from the atmosphere, Si and N in the liquid reach the supersaturated state, thus precipitating silicon nitride. During the reaction, the C in the liquid is also supersaturated, but at high temperatures of 1450 ℃, the SN is more stable and therefore does not form SiC. α-Si_3_N_4_ was selected due to its high thermal stability and high purity, which are advantageous for maintaining compositional consistency during processing.

**Fig. S2.** SEM images of silicon nitride nanowire networks.

Silicon nitride nanowires exhibit a three-dimensional interconnected network structure **(Fig. S2a)**, in which individual nanowires crosslink and entangle to form a spatially continuous skeleton. Such network architecture not only provides high structural integrity and load-transfer efficiency but also creates abundant interfacial contact regions for subsequent composite formation. The surface of each nanowire is relatively smooth and clean, without noticeable particulate attachments or surface contamination **(Fig. S2b)**, which may be beneficial for subsequent composite formation. The SN nanowire network benefits the PEKK composites by facilitating stress transfer, bridging microcracks, and deflecting crack propagation, thereby enhancing the mechanical integrity of the composites.

**Fig. S3** Microstructure characterization results of SN thin films: (a) TEM image and (b) high TEM image of SN film, (c) XRD spectra of SN (d, e) EDS elemental mapping of Si and N, (f) SAED pattern of SN

Transmission electron microscopy (TEM) images revealed that the surface of the silicon nitride nanowires (SN) was smooth and free of noticeable surface roughness or particulate attachments (**Fig. S3**a). High-resolution TEM (HRTEM) images clearly displayed lattice fringes with interplanar spacings of 0.67 nm and 0.43 nm, corresponding to the (100) and (101) planes of hexagonal α-Si₃N₄, respectively (Fig. S3b). A thin film sample was further analyzed by X-ray diffraction (XRD, **Fig. S3c**), confirming that the as-synthesized product was pure α-Si₃N₄. Distinct diffraction peaks were observed at 2θ = 20.6°, 22.9°, 31.0°, 34.6°, 35.3°, 38.9°, 43.4°, 57.7°, 62.4°, and 69.5°, which correspond to the (101), (110), (201), (102), (210), (211), (301), (222), (321), and (322) crystal planes of α-Si₃N₄ (PDF card No. 41-0360). These results support the successful synthesis of pure α-Si₃N₄ nanowire films.

Energy-dispersive X-ray spectroscopy (EDS) mapping of the SN nanowires (**Fig. S3d, e**) confirmed the homogeneous distribution of Si and N elements across the entire structure. The selected area electron diffraction (SAED) pattern exhibited distinct diffraction spots, indicating a highly ordered single-crystalline nature of the SN nanowires with minimal structural defects (**Fig. S3f**). Taken together, these comprehensive characterizations indicate that the synthesized SN nanowires possess a uniform morphology, pure α-phase composition, and well-defined crystalline structure.

**Fig. S4** Alizarin-Red staining of calcium nodules of the cells with samples at day 7, 14, and 21 after culturing;

**Fig. S5** Photographs of samples used for compression, flexural and tensile strength tests.

**Fig. S6** The 3D optical surface profile images of (a) PSN and (b) PSH.

The 3D surface profiles and surface roughness of the composites were recorded by a real color confocal microscope (LASERTEC, Japan). Surface roughness was comprehensively characterized using the related parameters, including Ra and Rq. Meanwhile, the roughness value of PSH (Ra=20.709 μm) is 14.7% higher than the roughness value of PSN (Ra=18.048 μm). The detailed 3D optical surface profile images of PSN and PSH are shown in **Fig. S6**.

1. *Corresponding author: E-mail: xijing_h@vip.tom.com
2. *Corresponding author: E-mail: zhangleilei@nwpu.edu.cn
3. *Corresponding author: E-mail: xjtumed1@163.com
